# Dynamic brain-body coupling of breath-by-breath O_2_-CO_2_ exchange ratio with resting state cerebral hemodynamic fluctuations

**DOI:** 10.1371/journal.pone.0238946

**Published:** 2020-09-21

**Authors:** Suk-tak Chan, Karleyton C. Evans, Tian-yue Song, Juliette Selb, Andre van der Kouwe, Bruce R. Rosen, Yong-ping Zheng, Andrew C. Ahn, Kenneth K. Kwong

**Affiliations:** 1 Department of Radiology, Athinoula A. Martinos Center for Biomedical Imaging, Massachusetts General Hospital, Charlestown, Massachusetts, United States of America; 2 Department of Psychiatry, Massachusetts General Hospital, Charlestown, Massachusetts, United States of America; 3 Department of Biomedical Engineering, The Hong Kong Polytechnic University, Hong Kong Special Administrative Region, China; Tokyo Joshi Ika Daigaku Toyo Igaku Kenkyujo Clinic, JAPAN

## Abstract

**Background:**

The origin of low frequency cerebral hemodynamic fluctuations (CHF) in the resting state remains unknown. Breath-by breath O_2_-CO_2_ exchange ratio (bER) has been reported to correlate with the cerebrovascular response to brief breath hold challenge at the frequency range of 0.008–0.03Hz in healthy adults. bER is defined as the ratio of the change in the partial pressure of oxygen (ΔPO_2_) to that of carbon dioxide (ΔPCO_2_) between end inspiration and end expiration. In this study, we aimed to investigate the contribution of respiratory gas exchange (RGE) metrics (bER, ΔPO_2_ and ΔPCO_2_) to low frequency CHF during spontaneous breathing.

**Methods:**

Twenty-two healthy adults were included. We used transcranial Doppler sonography to evaluate CHF by measuring the changes in cerebral blood flow velocity (ΔCBFv) in bilateral middle cerebral arteries. The regional CHF were mapped with blood oxygenation level dependent (ΔBOLD) signal changes using functional magnetic resonance imaging. Temporal features and frequency characteristics of RGE metrics during spontaneous breathing were examined, and the simultaneous measurements of RGE metrics and CHF (ΔCBFv and ΔBOLD) were studied for their correlation.

**Results:**

We found that the time courses of ΔPO_2_ and ΔPCO_2_ were interdependent but not redundant. The oscillations of RGE metrics were coherent with resting state CHF at the frequency range of 0.008–0.03Hz. Both bER and ΔPO_2_ were superior to ΔPCO_2_ in association with CHF while CHF could correlate more strongly with bER than with ΔPO_2_ in some brain regions. Brain regions with the strongest coupling between bER and ΔBOLD overlapped with many areas of default mode network including precuneus and posterior cingulate.

**Conclusion:**

Although the physiological mechanisms underlying the strong correlation between bER and CHF are unclear, our findings suggest the contribution of bER to low frequency resting state CHF, providing a novel insight of brain-body interaction via CHF and oscillations of RGE metrics.

## Introduction

Low frequency components (below 0.05 Hz) in the cerebral hemodynamic fluctuations (CHF) are used to characterize functional connectivity of cerebral resting state networks including the default mode network (DMN) [1, 2]. The origin of these low frequencies of CHF is still not clear, and many physiological candidates of cardiac and respiratory origin had been proposed contributing to this frequency bandwidth [3–6]. They include fluctuations related to respiratory variation (~0.03Hz) [3, 4], heart rate variability (0.05–0.15Hz) [5] and end-tidal carbon dioxide fluctuations (0–0.05Hz) [6].

Fluctuations of respiratory gas exchange (RGE) are of particular interest because RGE is a physiological process of removing carbon dioxide (CO_2_) as a metabolic waste from the blood and replenishing oxygen (O_2_) for energy consumption. Breath-by-breath RGE metrics include parameters like end-tidal partial pressures of O_2_ (P_ET_O_2_) and CO_2_ (P_ET_CO_2_), changes of the partial pressure of O_2_ (ΔPO_2_) and CO_2_ (ΔPCO_2_) [7, 8]. According to the concept of homeostasis, the homeostatic regulatory system is formed with arterial PO_2_ and PCO_2_ as regulated variables, peripheral and central chemoreceptors as sensors, brain stem as the control center, and diaphragm and respiratory muscles as effectors, to optimize systemic blood gases. While the detailed mechanisms between the change in central chemoreceptor activities and the change in cerebral blood flow (CBF) are topics of on-going research [9–11], arterial PO_2_ and PCO_2_ which are sensed by chemoreceptors are maintained within a range (or fluctuate) around the physiological ‘set point’ (i.e. mean) by the feedback control of diaphragm and respiratory muscles for ventilation in the homeostatic regulatory system. Spontaneous breathing is, therefore, part of a vital homeostatic process to optimize the systemic blood gases which can presumably regulate in turn CBF and O_2_ delivery to the brain [8, 12]. Such homeostatic fluctuations of arterial PO_2_ or PCO_2_ may interact synergistically and contribute to fluctuations of CHF during spontaneous breathing at rest.

RGE may be related to CHF because oscillations of RGE metrics (P_ET_O_2_, P_ET_CO_2_, respiratory exchange ratio) had been reported to be below 0.05Hz in humans [13, 14]. In our recent breath hold study, we made an emphasis on the ratio of ΔPO_2_ to ΔPCO_2_ between end inspiration and end expiration which we named breath-by-breath exchange ratio (bER), to quantify RGE as well [15]. We showed that cerebral hemodynamic responses to brief breath hold challenge were separately coupled with ΔPO_2_, ΔPCO_2_ and bER. It is interesting that bER was superior to either ΔPO_2_ or ΔPCO_2_ alone in coupling with the changes of global/regional cerebral hemodynamic response in our breath hold study. In this manuscript, we hypothesized a brain-body interaction during spontaneous breathing where various RGE metrics would separately contribute to CHF and the coupling of CHF with bER would be the strongest. Our study was different from previous works by Wise et al. [6] and Golestani et al. [16] which assumed that changes of PCO_2_ and PO_2_ were redundant.

The oscillations of P_ET_O_2_ and P_ET_CO_2_ during spontaneous breathing were shown to be non-redundant by at least three research teams [13, 17, 18] even though the arterial PO_2_ and PCO_2_ are expected to be correlated with each other through the natural RGE process. Interdependence but non-redundancy were also shown between P_ET_O_2_ and P_ET_CO_2_, between ΔPO_2_ and ΔPCO_2_, and between PO_2_ and PCO_2_ in healthy subjects under brief breath hold challenge [15]. Notably, had P_ET_O_2_/ΔPO_2_/PO_2_ and P_ET_CO_2_/ΔPCO_2_/PCO_2_ been redundant during spontaneous breathing, there would be no significant oscillations of respiratory exchange ratio as reported by Lenfant et al. [13]. The non-redundancy between P_ET_O_2_ and P_ET_CO_2_ [13, 17, 18] is highly significant because it challenges the long-held belief that the oscillations of the partial pressure of O_2_ (PO_2_) and CO_2_ (PCO_2_) are simply mirror image of each other [4], and encourages studying the separate effects of O_2_, CO_2_ and their ratio bER on CHF during spontaneous breathing.

In addition to the more commonly used P_ET_O_2_ and P_ET_CO_2_, we focused more on the contribution of RGE metrics including ΔPO_2_ and ΔPCO_2_ and bER in this manuscript. We preferred to use the ratio of ΔPO_2_/ΔPCO_2_ for the respiratory gas exchange instead of P_ET_CO_2_/P_ET_O_2_ with two reasons that we have explained with the findings in our recent breath hold study [15]. First, bER is a ratio which factors out the effects of ventilatory volume fluctuations [7] common to both ΔPO_2_ and ΔPCO_2_, but not to both P_ET_CO_2_ and P_ET_O_2_. The time courses of P_ET_CO_2_ and P_ET_O_2_ oscillate out of phase, while ΔPO_2_ oscillates in phase with ΔPCO_2_. Second, bER is a breath-by-breath dynamic form related to the steady-state respiratory exchange ratio (RER) described in the alveolar gas equation introduced by Fenn et al. [19]; the time-averaged value of bER over minutes is therefore mathematically equivalent to the reciprocal of RER. ΔPO_2_ and not P_ET_O_2_ is the designated term used in RER from the alveolar air equation [7, 19]. ΔPO_2_ is used as numerator in bER in this manuscript because of the strong positive correlation found previously between bER and cerebral hemodynamic responses under brief breath hold challenge [15]. RER has been used to evaluate resting systemic metabolic rate [20–22]. There are also technical differences between RER used in the literature and bER we used in this study as well as our previous breath hold study [15]. Traditionally, RER is derived by measuring the respiratory flow and the expired gases collected in Douglas bag connected to a closed circuit over several minutes. In our study, bER was derived by measuring the inspired and expired gases with a nasal tubing at each breath.

Given the dynamic changes in PO_2_ and PCO_2_ breath-by-breath in humans, we studied RGE metrics as surrogates of arterial blood gases with the recognition of the difference between respiratory metrics and blood gases. We studied the interaction between RGE metrics and CHF measured with transcranial Doppler sonography (TCD) and functional magnetic resonance imaging (fMRI). TCD has an advantage of acquiring data at high temporal resolution (~100 Hz), eliminating the concern of unwanted high frequency cardiovascular signal being aliased into the low frequency range which has a potential association with the minute-long oscillation of RGE metrics. The cerebral blood flow velocities (CBFv) were measured by TCD in the middle cerebral arteries (MCA) which supply most parts of the brain. The application of TCD allows the subjects to have measurements in an upright seated position, which is the usual position in respiratory physiology studies. Although TCD offers a high temporal resolution to evaluate CHF, it does not provide regional information. Regional mapping of spontaneous CHF was carried out instead with blood oxygen level-dependent (BOLD) signal changes measured by fMRI. We used BOLD-fMRI instead of arterial spin labeling (ASL) in MRI perfusion studies for two major reasons. ASL perfusion-related signal has a low contrast to noise ratio in comparison with the BOLD signal, especially in white matter area. ASL image acquisition at a temporal resolution of 4 seconds also under-samples the spontaneous fluctuations within the respiratory cycle of 4–6 seconds, which hampers the dynamic analyses between CHF and RGE metrics. While CBFv and BOLD signals are not equivalent surrogates for CBF, combining their strengths would be powerful to study the temporal and spatial features in the association between RGE and CHF.

In the present study, we aimed to evaluate the contribution of breath-by-breath RGE metrics to the CHF indicated by CBFv and BOLD signal changes during spontaneous breathing. To address the question of redundancy among RGE metrics during spontaneous breathing, we examined the correlations among bER, ΔPCO_2_, ΔPO_2_, P_ET_CO_2_ and P_ET_O_2_. In the TCD study, we measured the correlation of CBFv in the MCA with RGE metrics of bER, ΔPO_2_ and ΔPCO_2_. We also examined the temporal features and frequency characteristics of these RGE metrics and their coherence with CBFv. In the fMRI study, we mapped the association between regional BOLD signal changes and RGE metrics of bER, ΔPO_2_ and ΔPCO_2_. Brain regions showing a significant association between regional BOLD signal changes and RGE metrics were then compared with regions within the default mode network (DMN) outlined by the resting state connectivity analysis with the seed at the left precuneus. DMN encompasses brain regions responsible for the constant background activities at rest, showing higher metabolic and hemodynamic level than the other parts of the cortex [23]. The temporal features and frequency characteristics of these RGE metrics and their coherence with BOLD signal changes in the regions within DMN were also examined. The respiratory metric of respiration volume per unit time (RVT) was also derived to examine if the potential coherence between CHF and RGE metrics were resulting from respiratory variability. The investigation of the association between RGE metrics and CHF would provide new directions to the study of brain-body interaction, in addition to offering a physiological model to characterize the contribution of gas exchange elements in the low frequency resting state fluctuations, With the reference of cerebrovascular reactivity (CVR) to exogenous CO_2_ challenge, the potential of using the interaction between CHF and RGE metrics to evaluate CVR to the elements in spontaneous breathing was also discussed.

## Materials and methods

### Participants

Twenty-two volunteers aged from 19 to 48 years (mean age = 30.5 years, SD = 9.1 years, 14 males and 8 females) were included. Eleven of them participated in both TCD and MRI sessions, while the remaining participated in either one of the sessions. Subject demographics were shown in Table 1. All of them were recruited by e-mail and poster placement within the Partners hospital network. They were screened to exclude neurological, mental and medical disorders and drug abuse. TCD and MRI scanning were performed in the Athinoula A. Martinos Center for Biomedical Imaging at the Massachusetts General Hospital of Partners HealthCare. All the experimental procedures were explained to the subjects, and signed informed consent was obtained prior to the participation in the study. All components of this study were performed in compliance with the Declaration of Helsinki, and all procedures were approved by the Massachusetts General Hospital IRB of Partners HealthCare.

**Table 1:** Subject demographics and their participation in the TCD and MRI sessions.

Subjects	Gender	Age	TCD	MRI	
				Spontaneous Breathing	Exogenous Hypercapnic CO_2_ Challenge
s1	M	35	-	√	√
s2	M	48	-	√	√
s3	M	22	-	√	√
s4	M	46	-	√	-
s5	M	32	-	√	-
s6	M	29	-	√	-
s7	F	45	-	√	-
s8	M	19	-	√	-
s9	M	20	-	√	-
s10	M	38	√	√	√
s11	M	28	√	√	-
s12	M	27	√	√	√
s13	M	32	√	√	√
s14	M	22	√	√	√
s15	M	32	√	√	√
s16	F	26	√	√	√
s17	F	27	√	√	√
s18	F	47	√	√	-
s19	F	25	√	√	-
s20	F	23	√	√	-
s21	F	25	√	-	-
s22	F	23	√	-	-
Number of subjects:			13	20	10

Our study was divided into two parts: Part I and Part II. In Part I TCD study, we aimed to correlate the RGE metrics including bER, ΔPCO_2_ and ΔPO_2_ with CBFv in MCAs which serve as CHF in the major cerebral arterial supply during spontaneous breathing. We also examined the temporal features and frequency characteristics of these RGE metrics and their coherence with CBFv. Thirteen subjects participated in TCD sessions. In Part II MRI study, we aimed to map the association between these RGE metrics (bER, ΔPCO_2_ and ΔPO_2_) and BOLD signal changes which serve as regional CHF during spontaneous breathing. Twenty subjects participated in MRI sessions. Ten out of 20 subjects had additional exogenous CO_2_ challenge in the MRI sessions. Before we correlated the changes of RGE with CBFv and BOLD signal changes, we examined the correlations among the respiratory metrics (bER, ΔPCO_2_ and ΔPO_2_) acquired in both TCD and MRI sessions.

### Part 1: TCD

#### Transcranial Doppler scanning

Before the study of blood flow velocity in intracranial arteries, the subject was allowed to rest at least 20–30 minutes in an upright seated position for hemodynamic stabilization. The blood pressure measured in the subject was within the normal range of systolic blood pressure below 130 mmHg [24]. With the subject in an upright seated position, a dual probe setting with 2MHz transducers in conjunction with TCD system (Delicate EMS-9U, Shenzhen, China) was used for simultaneous recording of CBFv in the MCA on both left and right sides while the subject was at rest. Two transducers were attached to the left and right temporal bone windows by velcro. The depth of the Doppler samples was confined to the M1 segment, which is at the main stem of the MCA, for all the subjects.

A white crosshair in the black background was presented visually to the subject by a computer using the software Eprime Professional 2.0 (Psychology Software Tools, Inc., Pittsburgh, USA) to fixate their eye movement. The total duration of the CBFv data acquisition lasted 10 minutes.

Physiological changes including PCO_2_, PO_2_, electrocardiogram (ECG) and peripheral blood pressure were measured simultaneously with TCD acquisition. A biological amplifier (FE132, ADInstruments, Inc., CO, USA) and a finometer (Finapres Medical Systems, Netherlands) were used to measure ECG and peripheral blood pressure, respectively. A small nasal tubing was placed at the subject’s nostril to sample PCO_2_ and PO_2_ via gas analyzers (Capstar-100, Oxystar-100, CWE, Inc., PA, USA) after calibrating to the barometric pressure on the day of TCD session and correcting for vapor pressure. Small nasal tubing was chosen to sample PCO_2_ and PO_2_ because of several reasons. In the current study, we standardized all the physiological set-up in both TCD and MRI sessions. We did not measure PCO_2_ and PO_2_ from a facemask connected to a closed breathing circuit with non-rebreathing valves because of the space constraint of the MRI head coil, or from the expired gases collected in Douglas bag because of the slow sampling of gases in the units of minutes. Sampling gases with small nasal tubing has an advantage that the subjects breathe normally through the nose. In the gas sampling circuit, the same gas sample volume was used in both CO_2_ and O_2_ analyzers at the same gas sampling flow rate. The CBFv and physiological measurements were synchronized using trigger signals from E-prime. All the CBFv time series and physiological recordings were stored for offline data analysis.

### Part 2: MRI

#### MRI acquisition

MRI brain scanning was performed on a 3-Tesla scanner (Siemens Medical, Erlangen, Germany). The head was immobilized in a standard head coil with foam pads. The following whole brain MRI datasets were acquired on each subject: 1) standard high-resolution sagittal images acquired with volumetric T1-weighted 3D-MEMPRAGE (TR = 2530ms, TE = 1.74ms/3.6ms/5.46ms/7.32ms, flip angle = 7º, FOV = 256×256mm, matrix = 256×256, slice thickness = 1mm); 2) BOLD-fMRI images acquired with gradient-echo echo planar imaging (EPI) sequence (TR = 1450ms, TE = 30ms, flip angle = 90º, FOV = 220×220mm, matrix = 64×64, thickness = 5mm, slice gap = 1mm) while the subject was at rest. The visual presentation of the crosshair, the physiological set-up for the sampling of PCO_2_ and PO_2_ in the MRI session were the same as those used in the TCD session. The gas analyzers were again calibrated to the barometric pressure on the day of MRI session and corrected for vapor pressure. ECG was measured using a Siemens physiological monitoring unit (Siemens Medical, Erlangen, Germany). Physiological changes including PCO_2_, PO_2_, ECG and respiration were measured simultaneously with MRI acquisition. All the physiological measurements, including those of PCO_2_, PO_2_ and ECG were synchronized using trigger signals from the MRI scanner. BOLD-fMRI images and physiological recordings were stored for offline data analysis.

#### Exogenous CO2 challenge

Ten out of 20 subjects had additional exogenous CO_2_ challenge in the MRI sessions. An in-house gas delivery and mixing system comprising of a medical gas mixer in series with a manifold of flow meters was used to mix and deliver gas mixture for exogenous CO_2_ challenge. Given that there is significant inter-individual variance in resting P_ET_CO_2_ [25], resting P_ET_CO_2_ was assessed in subjects via calibrated capnograph before the exogenous CO_2_ challenge. The subject wore nose-clip and breathed through a mouth-piece on an MRI-compatible circuit designed to maintain the P_ET_CO_2_ within ± 1–2 mmHg of target P_ET_CO_2_ [26, 27]. The fraction of inspired carbon dioxide was adjusted to produce steady-state conditions of normocapnia and mild hypercapnia (4–8 mmHg above the subject’s resting P_ET_CO_2_). The CO_2_ challenge paradigm consisted of 2 consecutive phases (normocapnia and mild hypercapnia) repeating 6 times with 3 epochs of 4 mmHg increase and 3 epochs of 8 mmHg increase of P_ET_CO_2_. The normocapnic phase lasted 60–90 seconds, while the mild hypercapnic phase lasted 30 seconds. The same paradigm was also used and described in our other study [15]. The total duration of the exogenous CO_2_ hypercapnic challenge lasted 10 minutes.

When the subject had exogenous CO_2_ challenge in MRI session, BOLD-fMRI images were acquired using the same EPI sequence for resting state. The PCO_2_ and PO_2_ were sampled through the air filter connected with the mouthpiece, and the sampled gases were measured by calibrated gas analyzers. The respiratory flow was measured with respiratory flow head (MTL300L, ADInstruments, Inc., CO, USA) on the breathing circuit via calibrated spirometer (FE141, ADInstruments, Inc., CO, USA). The physiological measurements were synchronized with MRI images using trigger signals from the MRI scanner. All the BOLD-fMRI images and physiological recordings were stored for offline data analysis.

### Data analysis

#### Processing of physiological data

The physiological data from both TCD and MRI sessions were analyzed using Matlab R2014a (Mathworks, Inc., Natick, MA, USA). Technical delay of PCO_2_ and PO_2_ was corrected by cross-correlating the time series of PCO_2_ and PO_2_ with respiratory phases determined from the artifactual displacement due to chest excursion on ECG time series in the TCD sessions, with the respiratory phases from respiratory bellow or respiratory flow measured with respiratory flow head via spirometer in the MRI sessions.

End inspiration (I) and end expiration (E) were defined on the time series of PO_2_ and PCO_2_ (S1 Fig). The breath-by-breath P_ET_CO_2_ and P_ET_O_2_ were extracted at the end expiration of PCO_2_ and PO_2_ time series respectively. ΔPO_2_ is defined as (inspired PO_2_ –expired PO_2_) and ΔPCO_2_ is defined as (expired PCO_2_ –inspired PCO_2_). Breath-by-breath O_2_-CO_2_ exchange ratio (bER) is defined as the ratio of ΔPO_2_ to ΔPCO_2_ measured between end inspiration and end expiration at each breath, whereas RER at steady state from alveolar air equation introduced by Fenn et al. [19] is formulated as the ratio of ΔPCO_2_ to ΔPO_2_ over minutes. The product of ΔPO_2_ and ΔPCO_2_ was not used to evaluate the interaction with CHF because the effects of fluctuations due to ventilation would be exacerbated in ΔPO_2_×ΔPCO_2_ (S2 Fig).

Simple correlation analyses were applied to the time series of RGE metrics (bER, ΔPCO_2_, and ΔPO_2_) in pairs. The correlation was considered significant at p<0.05.

### Part 1

#### Preprocessing of CBFv data

The CBFv data were analyzed using Matlab R2014a (Mathworks, Inc., Natick, MA, USA). A median filter was applied to the data to reduce artifactual spikes. Beat-by-beat systolic peaks and end-diastolic troughs were determined using custom Matlab function and corrected on the graphical user interface incorporated in the function. Systolic peaks and diastolic troughs of cardiac cycles on the CBFv time series showing persistent artifacts were excluded in the following analysis. TCD data in both left and right MCAs were acquired on 13 subjects. One of the 13 TCD datasets had persistent artifacts in over one-third of the CBFv time series acquired in the LMCA, and another one had persistent artifacts in CBFv data acquired in RMCA. The CBFv time series of those particular TCD runs that showed persistent artifacts were excluded in further analysis, and the CBFv time series acquired in the contralateral MCA that did not show persistent artifacts were retained. Time series of mean CBFv were derived by integrating the CBFv over each cardiac cycle. To reduce the large inter-individual variations of absolute blood flow velocities [28, 29] and to remove the dependence of insonation angle [30], the percent change of CBFv (ΔCBFv) relative to baseline value in the left and right MCAs was derived. The mean CBFv for 30 seconds at the beginning of the time series was chosen as the baseline.

#### Correlation analyses between ΔCBFv and RGE metrics

The time series of ΔCBFv were correlated with those of bER, ΔPCO_2_ and ΔPO_2_ separately. The correlation indicated by Pearson's correlation coefficient was considered significant at p<0.05. Fisher’s Z-transformation was used to transform Pearson’s correlation coefficients to Fisher’s z scores for group analysis. Paired t-tests were used to compare the Fisher’s z scores representing the correlation between ΔCBFv and bER with those indicating the correlation between ΔCBFv and other physiological parameters besides bER. Differences were considered to be significant at p<0.05. We did not apply a low pass filter to CHF before our correlation analyses, even though applying a low pass filter to CHF is a standard preprocessing step in the resting state analysis [1].

#### Dynamic analysis of coherence between ΔCBFv and RGE metrics as a function of time and frequency

Wavelet transform coherence (WTC) was employed to demonstrate the dynamic interaction between ΔCBFv and RGE metrics (S3 Fig) in the time-frequency domain. WTC is a method for analyzing the coherence and phase lag between two time series as a function of both time and frequency [31, 32]. The temporal and phase information of WTC has been used to map the dynamic connectivity of the brain regions related to heart rate changes [33]. It is therefore well suited to investigate the dynamic changes in the coupling between the time series of ΔCBFv and RGE metrics including bER, ΔPCO_2_, and ΔPO_2_, as well as the phase lag of ΔCBFv to bER, ΔPCO_2_ and ΔPO_2_. We used the Matlab wavelet cross-spectrum toolbox developed by Grinsted et al. [32]. Squared wavelet coherence between the time series of each RGE metric and ΔCBFv was separately plotted with x-axis as time and y-axis as a scale which has been converted to its equivalent Fourier period. An example of squared wavelet coherence between bER and ΔCBFv in right MCA from a representative subject during spontaneous breathing is shown in S3B Fig. The magnitude of wavelet transform coherence ranged between 0 and 1 that can be conceptualized as a localized correlation coefficient in time and frequency space [32]. An arrow indicates the phase angle between the two time series, with bER leading ΔCBFv, at particular samples of the time-frequency plane: a rightward pointing arrow indicates that the time series are in phase, or positively correlated (ϕ= 0); a leftward pointing arrow indicates anti-correlation (ϕ = π), and the downward and upward pointing arrows indicate phase angles of π/2 and -π/2 relative to ϕ = 0, respectively. Areas inside the ‘cone of influence’, which are locations in the time-frequency plane where edge effects give rise to lower confidence in the computed values, are shown in faded color outside of the conical contour. The statistical significance level of the wavelet coherence is estimated using the Monte Carlo method, and the 5% significance level against red noise is shown as a thick contour in the squared wavelet coherence plot. The wavelet coherence magnitudes and phases bounded by thick contour outside the cone of influence are considered significant.

Time-averaged coherence is defined as the total significant coherence at each scale of Fourier periods (converted into frequency) where the wavelet coherence magnitude exceeded 95% significance level, normalized by the maximum possible coherence outside the cone of influence at that particular scale (S3C Fig). It is interpreted in a similar way as the coherence in the transfer function analysis which has been used in cerebral autoregulation study [34].

Interpreting the time-averaged coherence irrespective of phase lag raised the question that the coherence with positive correlation between two time series (at phase lag of 0±π/2) and the coherence with negative correlation (at phase lag of π±π/2) might be mixed together. Therefore, mean time-averaged coherence at the phase lags of 0±π/2 and π±π/2 were separately averaged across all the subjects who participated in the TCD sessions to explore the Fourier periods/frequency bandwidths that oscillations of ΔCBFv were in synchrony with the time series of each RGE metric (bER, ΔPCO_2_ and ΔPO_2_) when they were at rest.

### Part 2

#### Preprocessing of BOLD-fMRI data

All the BOLD-fMRI data were imported into the software Analysis of Functional NeuroImage (AFNI) [35] (National Institute of Mental Health, http://afni.nimh.nih.gov) for time-shift correction, motion correction, normalization and detrending. Details of preprocessing with AFNI are given as follows. The first 12 volumes in the first 12 time points of each functional dataset, collected before equilibrium magnetization was reached, were discarded. Each functional dataset was corrected for slice timing, motion-corrected and co-registered to the first image of the functional dataset using three-dimensional volume registration. Components of motion were removed from the co-registered functional dataset using orthogonal projection. The clean functional dataset was then normalized to its mean intensity value across the time-series. Voxels located within the ventricles and outside the brain defined in the parcellated brain volume using FreeSurfer [36, 37] (MGH/MIT/HMS Athinoula A. Martinos Center for Biomedical Imaging, Boston, http://surfer.nmr.mgh.harvard.edu) were excluded from the following analyses of functional images. Individual subject brain volumes with time series of percent BOLD signal changes (ΔBOLD) were derived.

#### Linear regression for the association between ΔBOLD and RGE metrics in individual subject

Linear regression analysis was used to evaluate the association between ΔBOLD, bER, ΔPO_2_ and ΔPCO_2_ for each subject. For each voxel of the preprocessed brain volume, the time series of ΔBOLD was separately regressed on bER, ΔPO_2_ and ΔPCO_2_. Regression coefficient beta (β) value was defined as the percent BOLD signal changes per unit change of the regressor (bER, ΔPO_2_ or ΔPCO_2_). Individual subject brain volumes with β magnitudes were registered onto their own anatomical scans and transformed to the standardized space of Talairach and Tournoux [38]. Monte Carlo simulation was used to correct for multiple comparisons [39]. To protect against type I error, individual voxel probability threshold of p<0.005 was held to correct the overall significance level to α<0.05. Based upon a Monte Carlo simulation with 2000 iteration processed with ClustSim program [40], it was estimated that a 476mm^3^ contiguous volume would provide the significance level α<0.05, which met the overall corrected threshold of p<0.05.

#### Group region-of-interest (ROI) analysis for the association between ΔBOLD and RGE metrics

For each subject who participated in MRI scanning, β magnitude values derived by regressing ΔBOLD on bER (β_bER_), ΔBOLD on ΔPO_2_ (β_ΔPO2_) and ΔBOLD on ΔPCO_2_ (β_ΔPCO2_) were separately averaged in each of the 160 brain regions parcellated by the software FreeSurfer. One-sample t-tests were used to analyze regional β_bER_, β_ΔPO2_ and β_ΔPCO2_ in the subject group. False discovery rate was used to correct for multiple comparisons [41, 42]. Significant association in group was considered at false discovery rate adjusted p_fdr_<0.05.

For each brain region, we also calculated the number of voxels with significant ΔBOLD that reached cluster-corrected threshold p<0.05 in the individual subject analysis. The number of voxels with significant ΔBOLD were then normalized to the total number of voxels in each brain region. The percentage of voxels in each brain region with significant ΔBOLD, namely voxelβ_bER_, voxelβ_ΔPO2_ and voxelβ_ΔPCO2_, for RGE metrics bER, ΔPO_2_ and ΔPCO_2_ were calculated respectively. Individual subject brain volumes with regional voxelβ due to bER (voxelβ_bER_), ΔPO_2_ (voxelβ_ΔPO2_) and ΔPCO_2_ (voxelβ_ΔPCO2_) were obtained. One-sample t-tests were used to analyze regional voxelβ_bER_, voxelβ_ΔPO2_ and voxelβ_ΔPCO2_ in the subject group. False discovery rate was used to correct for multiple comparisons [41, 42]. Significant association in group was considered at p_fdr_<0.05.

In the group analysis, we preferred the ROI analysis of 160 brain regions for β_bER_, β_ΔPO2_ and β_ΔPCO2_ instead of the commonly used voxel-by-voxel analysis because the maps of β values were compared side-by-side with the maps of voxelβ_bER_, voxelβ_ΔPO2_ and voxelβ_ΔPCO2_ which require the ROI analysis to evaluate the percentage of voxels in each brain region with significant ΔBOLD.

#### Group maps of regional ΔBOLD associated with RGE metrics vs. group functional connectivity maps with the seed at left precuneus

Seed-based analysis with the seed at the left precuneus was used to generate the functional connectivity map for an individual subject. For each subject, after preprocessing of BOLD data, a low pass filtering at 0.03Hz was applied onto the brain volume with time series of ΔBOLD. The time series of ΔBOLD in the voxels within left precuneus were averaged. The averaged ΔBOLD time series of left precuneus was correlated with the ΔBOLD time series of each voxel in the brain volume. Pearson's correlation coefficient was used to indicate the strength of correlation and converted to z scores using the Fisher’s Z transformation. For the comparison with the regional β and voxelβ maps, the Fisher’s z scores in each of the 160 brain regions were averaged for each subject. Individual subject brain volumes with regional Fisher's z scores from all the subjects who participated in MRI sessions were subjected to group analysis using a one-sample t-test. False discovery rate was again used to correct for multiple comparisons [39, 41, 42]. Significant correlation in group was considered at p_fdr_<0.05.

#### Dynamic analysis of coherence between bER and ΔBOLD in brain regions within DMN

To further verify the dynamic coupling between bER and CHF in DMN, we used the WTC method to examine the dynamic changes in coupling between the time series of RGE metrics (bER, ΔPCO_2_, and ΔPO_2_) and ΔBOLD in three brain regions within DMN. They included inferior parietal lobule (IPL), posterior cingulate (PCC) and precuneus (PCun). The analysis procedures were the same as those described for the dynamic analysis of coherence between ΔCBFv and RGE metrics. The mean time-averaged coherence between each RGE metric and ΔBOLD from each of the three brain regions at the phase lag of 0±π/2 was averaged across all the subjects who participated in MRI sessions. Similarly, the mean time-averaged coherence between each RGE metric and ΔBOLD from each of the three brain regions at the phase lags of π±π/2 were averaged across the same group of the subjects.

#### Dynamic analysis of coherence between respiratory volume per unit time (RVT) and ΔBOLD in brain regions within DMN

To confirm that the respiratory-variation-related fluctuations reported by Birn et al. [3] had little contribution to CHF within the DMN, we used the same WTC method to examine the dynamic changes in coupling between the time series of RVT (respiration volume per time) and ΔBOLD in three brain regions within DMN (IPL, PCC and PCun). Ten out of 20 subjects had RVT measurements with a respiratory bellow. According to the method described by Birn et al. [3], RVT was computed by the difference between the maximum and minimum bellow positions at the peaks of inspiration and expiration respectively, and this difference was then divided by the period of the respiratory cycle. The mean time-averaged coherence between RVT and ΔBOLD from each of the three brain regions at the phase lag of 0±π/2 were averaged across 10 subjects. Similarly, the mean time-averaged coherence between RVT and ΔBOLD from each of the three brain regions at the phase lag of π±π/2 were averaged across the same group of subjects.

#### Regional CVR quantification under exogenous CO_2_ challenge

Linear regression analysis was used to derive CVR from the time series of ΔBOLD and vasoactive stimulus when the subject was under exogenous CO_2_ challenge. We used the time series of P_ET_CO_2_ a regressor in a linear regression analysis. CVR under exogenous CO_2_ challenge was defined as the percent BOLD signal changes per mmHg change of P_ET_CO_2_. Therefore CVR was quantified by the coefficient of regression, i.e. the slope.

For each subject who participated in exogenous CO_2_ MRI scanning, CVR values derived from regressing ΔBOLD on P_ET_CO_2_ (CVR_CO2-PETCO2_) were separately averaged in each of the 160 brain regions parcellated by the software FreeSurfer. To study the CVR in group, one-sample t-tests were applied onto the brain volumes with regional CVR_CO2-PETCO2_. False discovery rate was used to correct for multiple comparisons.

## Results

Subject demographics are shown in Table 1. The mean values and standard deviation of the time series of P_ET_CO_2_, P_ET_O_2_, ΔPCO_2_, ΔPO_2_ and bER measured in the TCD and MRI sessions are summarized in Table 2.

**Table 2:** RGE metrics in TCD and MRI sessions.

	TCD					BOLD				
Subjects	P_ET_CO_2_ (mmHg)	P_ET_O_2_ (mmHg)	ΔPCO_2_ (mmHg)	ΔPO_2_ (mmHg)	bER	P_ET_CO_2_ (mmHg)	P_ET_O_2_ (mmHg)	ΔPCO_2_ (mmHg)	ΔPO_2_ (mmHg)	bER
s1	---	---	---	---	---	42.6 (2.1)	99.4 (5.0)	42.3 (2.3)	52.6 (6.2)	1.2 (0.1)
s2	---	---	---	---	---	38.3 (1.3)	111.5 (1.0)	36.5 (2.0)	35.6 (1.4)	1.0 (0.1)
s3	---	---	---	---	---	35.7 (3.8)	109.0 (3.6)	35.4 (4.4)	41.7 (4.3)	1.2 (0.2)
s4	---	---	---	---	---	35.4 (1.2)	114.5 (2.1)	33.0 (1.2)	37.1 (2.6)	1.1 (0.1)
s5	---	---	---	---	---	45.7 (2.1)	113.9 (3.4)	42.1 (3.2)	29.4 (3.8)	0.7 (0.1)
s6	---	---	---	---	---	33.8 (4.0)	118.8 (4.8)	30.5 (4.0)	25.1 (5.3)	0.8 (0.1)
s7	---	---	---	---	---	38.9 (1.8)	116.2 (4.6)	33.5 (1.7)	30.8 (5.3)	0.9 (0.1)
s8	---	---	---	---	---	29.0 (1.8)	103.9 (4.3)	27.7 (1.9)	50.9 (5.2)	1.8 (0.1)
s9	---	---	---	---	---	38.3 (1.3)	98.0 (2.5)	38.1 (1.5)	55.3 (3.0)	1.5 (0.1)
s10	39.1 (1.5)	107.3 (3.2)	39.4 (1.5)	44.3 (3.7)	1.1 (0.1)	41.4 (1.8)	103.8 (3.2)	40.1 (1.8)	43.0 (3.8)	1.1 (0.1)
s11	39.1 (2.7)	113.9 (4.1)	38.5 (2.8)	44.1 (4.6)	1.1 (0.1)	33.0 (2.9)	111.1 (5.3)	32.3 (3.0)	37.1 (6.1)	1.1 (0.1)
s12	30.6 (3.0)	119.4 (6.2)	29.5 (3.0)	30.8 (7.0)	1.0 (0.1)	38.9 (2.5)	107.8 (4.0)	37.8 (2.6)	39.6 (4.6)	1.0 (0.1)
s13	37.2 (0.8)	107.3 (2.0)	37.3 (0.9)	43.1 (2.5)	1.2 (0.1)	40.1 (0.9)	108.1 (3.5)	38.5 (1.0)	41.9 (4.3)	1.1 (0.1)
s14	39.4 (1.5)	100.9 (3.2)	38.3 (1.8)	49.7 (4.0)	1.3 (0.1)	36.6 (0.7)	108.7 (1.4)	36.0 (0.8)	40.0 (1.8)	1.1 (0.0)
s15	34.7 (2.8)	106.4 (4.1)	34.9 (2.9)	45.8 (4.6)	1.3 (0.1)	36.9 (1.9)	114.4 (2.5)	35.6 (1.9)	34.9 (2.7)	1.0 (0.0)
s16	25.9 (3.2)	123.8 (4.5)	26.0 (3.3)	29.3 (5.2)	1.1 (0.1)	34.6 (1.3)	117.8 (2.6)	32.4 (1.4)	34.5 (3.1)	1.1 (0.1)
s17	35.9 (0.5)	109.1 (1.2)	35.3 (0.5)	41.5 (1.4)	1.2 (0.0)	35.0 (0.6)	112.2 (1.6)	34.1 (0.7)	38.8 (1.9)	1.1 (0.0)
s18	34.0 (1.4)	117.1 (3.9)	33.4 (1.4)	33.4 (4.6)	1.0 (0.1)	35.4 (3.2)	115.3 (10.8)	33.8 (3.3)	38.5 (12.9)	1.1 (0.3)
s19	33.9 (0.8)	118.2 (2.2)	29.3 (1.0)	34.6 (2.9)	1.2 (0.1)	36.7 (1.0)	113.5 (2.2)	32.2 (1.1)	39.2 (2.4)	1.2 (0.1)
s20	32.4 (2.1)	116.6 (3.1)	29.1 (2.6)	35.5 (4.0)	1.2 (0.1)	34.2 (1.9)	114.4 (4.0)	30.2 (2.1)	38.6 (4.8)	1.3 (0.1)
s21	36.1 (4.5)	126.4 (4.9)	28.9 (6.8)	21.6 (6.5)	0.7 (0.1)	---	---	---	---	---
s22	32.4 (1.7)	117.5 (2.8)	28.1 (2.1)	34.1 (3.5)	1.2 (0.1)	---	---	---	---	---

Mean values (SD) of P_ET_CO_2_, P_ET_O_2_, ΔPCO_2_, ΔPO_2_ and bER for all subjects who participated in the TCD sessions (n = 13) (*left*), and for those who participated in the MRI sessions (n = 20) (*right*).

### Correlation among bER, ΔPO_2_ and ΔPCO_2_

**Fig 1:**
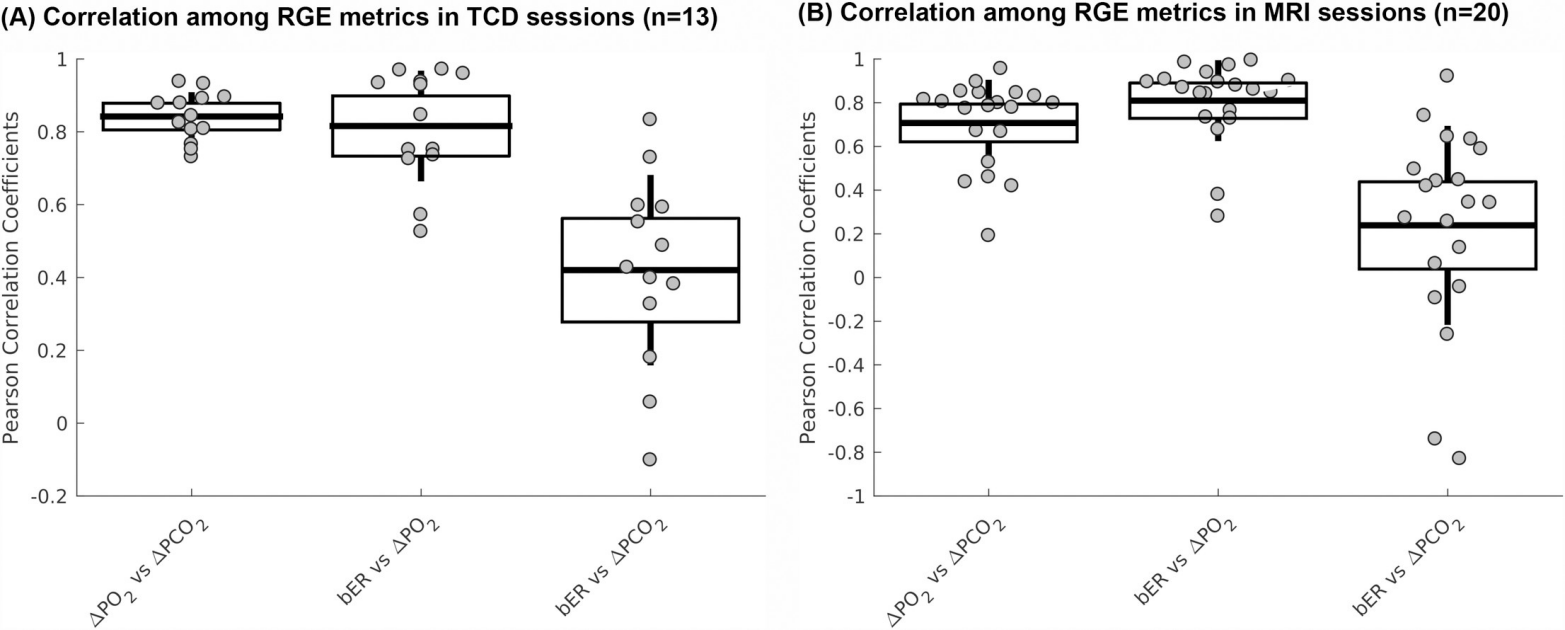
Correlation among breath-by-breath RGE metrics during spontaneous breathing. (A) Correlations among breath-by breath RGE metrics (bER, ΔPO_2_ and ΔPCO_2_) in all subjects who participated in TCD sessions, and (B) those who participated in MRI sessions. Each gray circle represents the Pearson’s correlation coefficient from the correlation analysis of the time series of parameter pair shown on the x-axis for each subject. The thick middle horizontal line, the box and the vertical rod represent the mean, 95% confidence interval and standard deviation of the group data, respectively.

The correlations among the RGE metrics (bER, ΔPO_2_ and ΔPCO_2_) in TCD and MRI sessions are shown in Fig 1 and summarized in S1 Table. Correlation coefficients between ΔPO_2_ and ΔPCO_2_ ranged from 0.7 to 0.9 in TCD sessions and from 0.2 to 0.9 in MRI sessions, demonstrating that ΔPO_2_ and ΔPCO_2_ are not necessarily redundant in either TCD or MRI sessions. The range of correlation strength between ΔPO_2_ and ΔPCO_2_ in the MRI sessions was larger than that in TCD sessions. Correlation between bER and ΔPO_2_ was stronger than that between bER and ΔPCO_2_.

### Part 1

#### Correlation between ΔCBFv and RGE metrics

**Fig 2:**
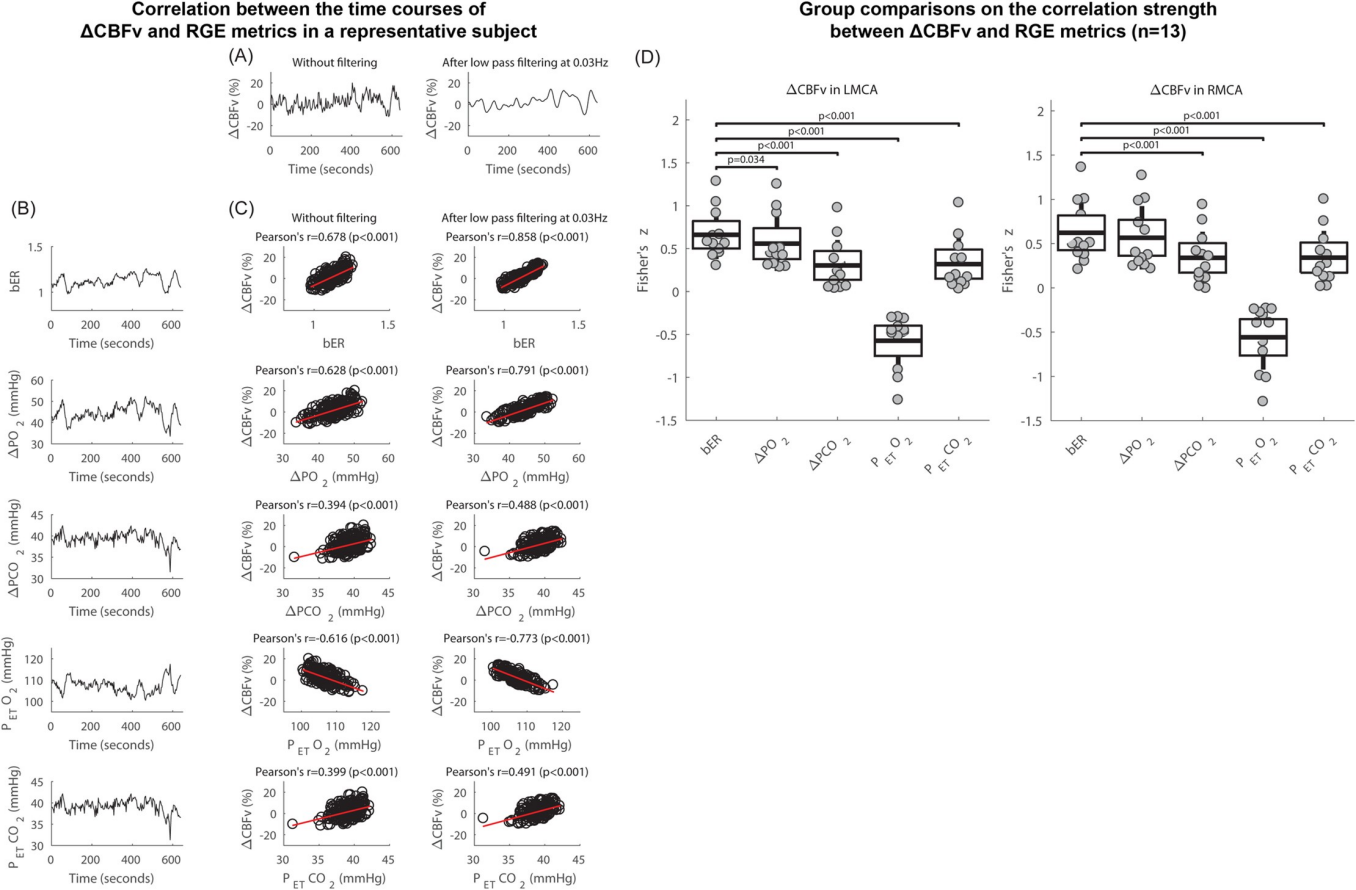
Correlation between the time series of ΔCBFv and RGE metrics during spontaneous breathing. (A) Time series of ΔCBFv in right MCA of a representative subject at rest, without filtering and after applying low pass filter at 0.03Hz. (B) Time series of RGE metrics of bER, ΔPO_2_, ΔPCO_2_, P_ET_O_2_ and P_ET_CO_2_ acquired simultaneously with ΔCBFv of the same subject in (A). (C) Correlation between ΔCBFv without filtering and the RGE metrics (*left*), and that between ΔCBFv with low pass filtering at 0.03Hz and RGE metrics (*right*) of the same subject in (A). (D) For all 13 subjects who participated in TCD sessions, paired comparisons of Fisher's z scores between ΔCBFv in left MCA and bER, and those between ΔCBFv in left MCA and other RGE metrics besides bER (*left*), and the paired comparisons of Fisher's z scores between ΔCBFv in right MCA and bER, and those between ΔCBFv in right MCA and other RGE metrics besides bER (*right*). Each gray circle represents Fisher's z score from the correlation analysis of ΔCBFv and the parameter shown on the x-axis for each subject. The thick middle horizontal line, the box and the vertical rod represent the mean, 95% confidence interval and standard deviation of the group data, respectively.

The time series of ΔCBFv in the right MCA of a representative subject during spontaneous breathing with and without applying low pass filtering at 0.03Hz are shown in Fig 2A. The time series of bER, ΔPO_2_, ΔPCO_2_, P_ET_CO_2_ and P_ET_O_2_ acquired simultaneously with ΔCBFv for the same subject are shown in Fig 2B. All the time series including CBFv, bER, ΔPO_2_, ΔPCO_2_, P_ET_CO_2_ and P_ET_O_2_ of the representative subject showed prominent oscillations with periods of 0.5 to 2 minutes which is equivalent to the frequency range of 0.008Hz to 0.03Hz. Oscillations of bER, ΔPO_2_, ΔPCO_2_, P_ET_CO_2_ were in phase with CBFv, while the oscillations of P_ET_O_2_ were out of phase. Oscillation amplitude of ΔPO_2_ could double that of ΔPCO_2_ (Fig 2B), where the standard deviation values indicating variations over the ΔPO_2_ time series were larger than those over the ΔPCO_2_ time series as shown in Table 2.

With the simple correlation analyses, Fig 2C shows that the correlation of bER with ΔCBFv was the strongest among all the RGE metrics for the representative subject. ΔPO_2_ followed relatively closely behind bER, and ΔPCO_2_ was the weakest in correlation with ΔCBFv. ΔPCO_2_ and P_ET_CO_2_ shared very similar correlation results with ΔCBFv. The correlation coefficients between ΔCBFv and RGE metrics (bER, ΔPO_2_, ΔPCO_2_ and P_ET_CO_2_) for all 13 subjects who participated in TCD sessions are shown in S2 Table. The similar correlation of bER and ΔPO_2_ with ΔCBFv showed that ΔPO_2_ plays an essential role in bER correlation with CHF.

Applying a low pass filter of 0.03Hz to the time series of ΔCBFv (Fig 2A, right panel) increased the strength of correlation of CBFv with bER, ΔPO_2_ and ΔPCO_2_ (Fig 2C, right panel) but did not change the order of which RGE metric was more correlated with ΔCBFv.

In the group analysis, paired comparisons of Fisher’s z scores transformed from Pearson's correlation coefficients between ΔCBFv and RGE metrics for all 13 subjects are shown in Fig 2D and S2 Table. The Fisher’s z scores of the correlation between ΔCBFv and bER were used as a reference to compare with those of the correlation between ΔCBFv and other RGE metrics beside bER. ΔCBFv consistently showed a stronger correlation with bER and ΔPO_2_ than with ΔPCO_2_ and P_ET_CO_2_. The correlation of ΔCBFv in LMCA with bER was significantly stronger than that with ΔPO_2_, while the difference between the correlation of ΔCBFv in RMCA with bER and that with ΔPO_2_ did not reach statistical significance.

The differences revealed in paired comparisons again demonstrated that ΔPO_2_ and ΔPCO_2_ were not necessarily redundant.

#### Dynamic coherence between ΔCBFv and RGE metrics

**Fig 3:**
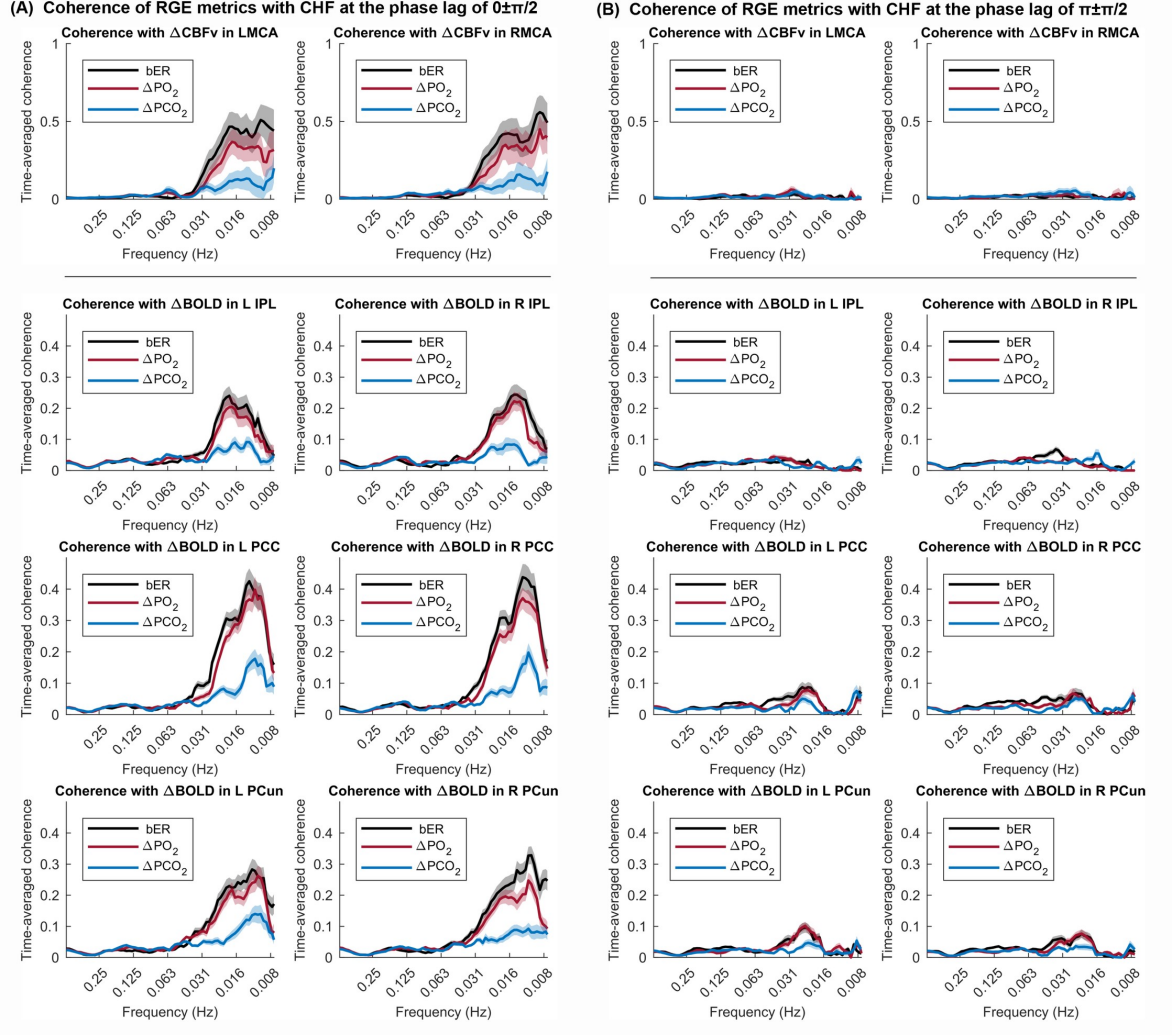
Time-averaged coherence between time series of RGE metrics and CHF. The mean time-averaged coherence in the frequency bandwidths from 0.008 to 0.25Hz (A) at the phase lag of 0±π/2, and (B) at the phase lag of π±π/2, were plotted (thick color lines). Color shaded areas represent standard error of the mean. Coherence between two time series at the phase lag of 0±π/2 indicates a positive correlation, while a negative correlation is represented by the coherence at the phase lag of π±π/2. Top panel shows the coherence of RGE metrics with ΔCBFv in LMCA and RMCA in TCD sessions (n = 13). The lower panel shows the coherence between RGE metrics and ΔBOLD in the inferior parietal lobule (IPL), posterior cingulate (PCC) and precuneus (PCun) within DMN in MRI sessions (n = 20).

The time-averaged coherence analyzed by WTC at the phase lag of 0±π/2 which indicates a positive correlation, and at the phase lag of π±π/2 which indicates a negative correlation, were plotted for all 13 subjects participated in TCD sessions (Fig 3). At the phase lag of 0±π/2, the mean time-averaged coherence between bER and ΔCBFv and that between ΔPO_2_ and ΔCBFv reached a value of 0.5 or above at the frequency range between 0.008 and 0.03Hz, while the mean time-averaged coherence between ΔPCO_2_ and ΔCBFv stayed below 0.1 (Fig 3A, top row). At the phase lag of π±π/2, the mean time-averaged coherence of all three RGE metrics with ΔCBFv stayed below 0.1 at the frequency range between 0.008 and 0.03Hz (Fig 3B, top row).

### Part 2

#### Association between ΔBOLD and RGE metrics in different brain regions

Brain maps on the association between ΔBOLD and RGE metrics (bER, ΔPO_2_ and ΔPCO_2_) during spontaneous breathing in a representative subject are shown in S4A Fig. The regional β magnitudes, namely ΔBOLD per unit change of each RGE metric in different brain regions, were mapped for bER (β_bER_), ΔPO_2_ (β_ΔPO2_) and ΔPCO_2_ (β_ΔPCO2_) (S4A Fig). Comparing with the maps of β_ΔPO2_ or β_bER_, the map of β_ΔPCO2_ showed fewer pixels which reached statistical significance (corrected p<0.05).

**Fig 4:**
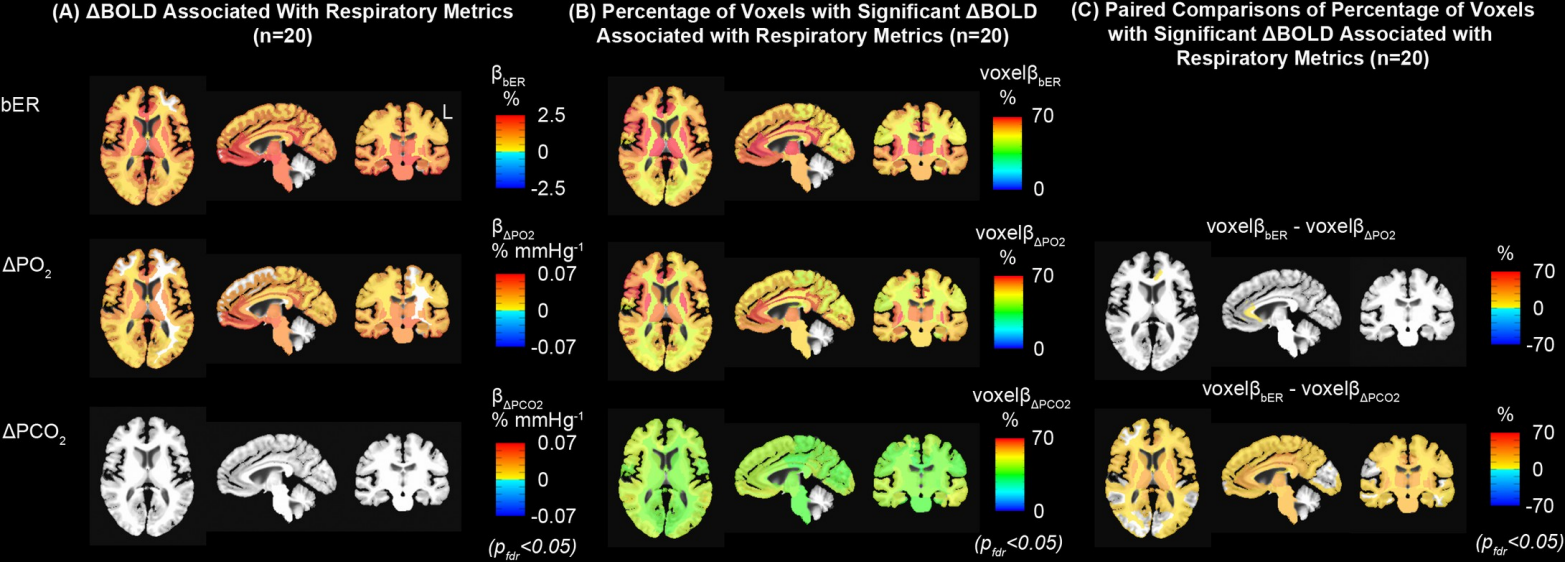
Regional association between ΔBOLD and RGE metrics in the MRI sessions. (A) Group maps showing the regional ΔBOLD per unit change of bER, ΔPO_2_ and ΔPCO_2_ (β_bER_, β_ΔPO2_ and β_ΔPCO2_). (B) Group maps showing the regional percentage of voxels with significant ΔBOLD per unit change of bER, ΔPO_2_ and ΔPCO_2_ (voxel_βbER_, voxelβ_ΔPO2_ and voxelβ_ΔPCO2_). (C) Paired comparisons of voxelβ maps. All the maps had been corrected for p_fdr_<0.05.

In addition to the brain maps from the individual subject, group regional β magnitudes were mapped for bER (β_bER_), ΔPO_2_ (β_ΔPO2_) and ΔPCO_2_ (β_ΔPCO2_) for all 20 subjects who participated in MRI sessions (S4B Fig and Fig 4A). ΔBOLD was found to be significantly associated with bER or ΔPO_2_ in most of the brain regions (Fig 4A) while association found between ΔBOLD and ΔPCO_2_ in a few brain regions (insula, medial orbitofrontal and temporal) (S4B Fig) did not reach statistical significance after correcting for multiple comparisons (Fig 4A).

In addition to using the β magnitude, brain volumes with the regional percentage of voxels having significant ΔBOLD for the regressors bER (voxelβ_bER_), ΔPO_2_ (voxelβ_ΔPO2_) and ΔPCO_2_ (voxelβ_ΔPCO2_) were analyzed in the same group of 20 subjects. Significant association of ΔBOLD with bER and ΔPO_2_ was shown in more than 50% of the voxels in the gray matter regions (Fig 4B), while the percentage of significant voxels was smaller in white matter regions. A smaller percentage of gray and white matter voxels showed a significant association of ΔBOLD with ΔPCO_2_. The maps of paired comparison between voxelβ_bER_ and voxelβ_ΔPO2_ showed significant difference in the percentage of significant voxels (Fig 4C) in the rostrum and genu of the left corpus callosum. However, the paired comparison between voxelβ_bER_ and voxelβ_ΔPCO2_ showed a significant difference in the percentage of significant voxels in many areas, including subcortical brain regions, insula and brainstem.

#### Regional association between bER and ΔBOLD overlapped with many areas of the default mode network

**Fig 5:**
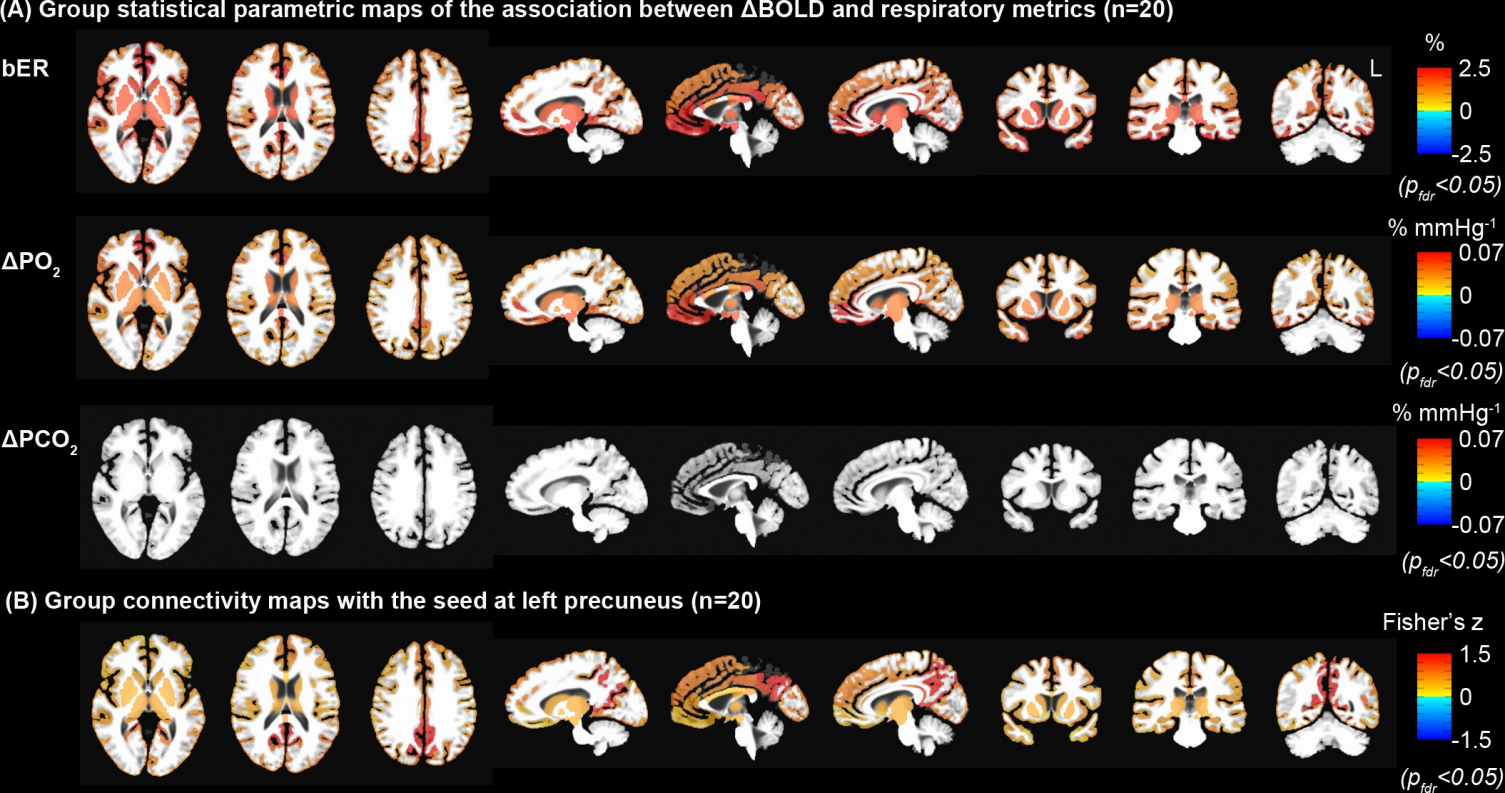
Comparison of statistical parametric maps derived from regression and connectivity analyses. (A) Group statistical parametric maps of the association between ΔBOLD and RGE metrics (bER, ΔPO_2_ and ΔPCO_2_). The white matter was excluded for the comparison with the group connectivity map. (B) Group connectivity map with the seed at left precuneus. ΔPCO_2_ was demonstrated to be much weaker than bER and ΔPO_2_ in its association with ΔBOLD in the brain regions of DMN. Comparing the top and bottom rows, similar regions of DMN were outlined with two independent methods of bER-CHF coupling and seed-based connectivity analysis. All the maps had been corrected for p_fdr_<0.05.

Group statistical parametric maps of regional association between ΔBOLD and RGE metrics in gray matter and brainstem (Fig 5A) were used to compare with the group connectivity map with the seed at left precuneus (Fig 5B) for the same group of 20 subjects in the MRI study. Brain regions showing significant association of ΔBOLD with bER and ΔPO_2_ included precuneus, posterior cingulate, anterior insula, caudate nucleus, superior temporal and inferior parietal regions. These brain regions overlapped with those in DMN reported by Raichle et al. [23] as well as those reported by Yeo et al. [43] and Choi et al. [44]. Additional regions such as posterior insula, putamen and occipital regions found in our group statistical parametric maps were also shown in the findings by Raichle et al. [23].

Group analysis of individual connectivity maps demonstrated significant increased connectivity in brain regions similar to the regions showing a significant association between ΔBOLD and bER (Fig 5). The close match between Fig 5A and 5B from the same BOLD datasets but acquired with independent methods supports that bER is capable of outlining the connectivity among brain regions in the DMN.

#### Dynamic coherence between bER and ΔBOLD in brain regions within DMN

We used WTC analysis to verify the dynamic coupling between bER and BOLD signal fluctuations in DMN regions such as left and right inferior parietal lobule (IPL), left and right posterior cingulate (PCC), and left and right precuneus (PCun) in all 20 subjects participated in MRI sessions. At the phase lag of 0±π/2, we showed that the mean time-averaged coherence between bER and ΔBOLD and that between ΔPO_2_ and ΔBOLD reached a value of 0.2 or above in general (Fig 3A, lower panel) at the frequency range between 0.008 and 0.03Hz, while the mean time-averaged coherence between ΔPCO_2_ and ΔBOLD peaked around 0.2 or below (Fig 3A, lower panel). At the phase lag of π±π/2, the mean time-averaged coherence of all three RGE metrics with ΔBOLD stayed below 0.1 at the frequency range between 0.008 and 0.03Hz (Fig 3B, lower panel).

#### Dynamic coherence between RVT and ΔBOLD in brain regions within DMN

To confirm that the RVT had little contribution to CHF within the DMN, we used the WTC method to examine the dynamic coupling between RVT and ΔBOLD extracted from IPL, PCC and PCun in 10 subjects who had their respiratory tracings measured by a respiratory bellow. We showed that the mean time-averaged coherence between RVT and ΔBOLD at the phase lag of 0±π/2 increased between 0.016 and 0.031Hz, and reduced at the frequency below 0.016Hz where the mean time-averaged coherence started to increase at the phase lag of π±π/2 (S5 Fig). The frequency distribution of coherence of CHF with RVT is shown to be very different from that with bER.

#### Regional CVR maps under exogenous CO_2_ challenge and during spontaneous breathing

Under exogenous CO_2_ challenge, most of the brain regions showed increased CVR_CO2-PETCO2_ in the subject group, especially thalamus, insula and putamen (S6 Fig). During spontaneous breathing, increased β_bER_ was found in most of the brain regions, while no significant association indicated by β_ΔPCO2_ were shown in most of the brain regions. The CVR map during spontaneous breathing indicated by β_bER_ resembled the CVR map under exogenous CO_2_ challenge indicated by CVR_CO2-PETCO2_.

## Discussion

There has been no previous study on the dynamic interactions between low frequency fluctuations of resting state CHF (ΔCBFv and ΔBOLD) and those of all three RGE metrics of bER, ΔPO_2_ and ΔPCO_2_. However, results in this study support such interactions even though the physiological mechanisms are still unclear. The time series of ΔPO_2_ and ΔPCO_2_ were shown to be interdependent but non-redundant. The prominent oscillations of bER, ΔPO_2_ and ΔPCO_2_ were characterized by periods of 0.5 to 2 minutes (0.008–0.03Hz) and were coherent with CHF in this frequency range. The coherence of CHF with bER or with ΔPO_2_ was much stronger than the coherence of CHF with ΔPCO_2_ in the frequency range between 0.008Hz and 0.03Hz. bER was shown in some brain regions to be superior to ΔPO_2_ in the association with CHF. Brain regions with the strongest bER-CHF coupling overlapped with many areas of DMN. Taken together, our study provides evidence that resting state CHF at low frequency show the strongest association with fluctuations attributed to bER.

### Oscillations of ΔPO_2_ and ΔPCO_2_ are interdependent but not necessarily redundant

The ΔPO_2_ and ΔPCO_2_ were often considered to be redundant [4, 6, 16]. In the present study, the average value of the correlation strength of around 0.8 and the relatively large spread of the correlation between ΔPO_2_ and ΔPCO_2_ demonstrated that while ΔPO_2_ and ΔPCO_2_ were interdependent they were not redundant to each other (Fig 1). Respiratory data during spontaneous breathing in healthy infants and adults were consistent with ours, showing that P_ET_O_2_ and P_ET_CO_2_ were correlated but not redundant [13, 17, 18].

The interdependent but non-redundant oscillations of ΔPO_2_ and ΔPCO_2_ during spontaneous breathing at the normoxic level are also supported by the findings of feedback loops involving the interaction among chemoreceptors, and blood gases demonstrated in many earlier studies on animal models [10, 45–48]. Most of these studies on the role of PO_2_ to stimulate peripheral chemoreceptors at the carotid body had been focused on hypoxia at PO_2_ level less than 60 mmHg [10, 49] where chemoreceptor activities rose quickly in a hyperbolic fashion. However, Biscoe et al. [45] showed that peripheral chemoreceptor activities were present from normoxia to hyperoxia up to arterial PO_2_ level of 190 mmHg and beyond. Lahiri et al. [48] reported that the stimulus thresholds of arterial PO_2_ and PCO_2_ for peripheral chemoreceptors were largely interdependent under normoxic conditions. A drop in arterial PO_2_ was routinely accompanied by increased chemoreceptor activities as well as an enhanced sensitivity of carotid chemoreceptors to arterial PCO_2_. While research on the mechanisms of interaction between peripheral and central chemoreceptors to optimize systemic blood gases is on-going [9–11], the chemoreceptor activities reported in these studies highlight the interdependence of PO_2_ and PCO_2_ which work synergistically to regulate the blood supply to the brain. bER can be one of the appropriate metrics to be used in the study of such synergism between ΔPO_2_ and ΔPCO_2_. The superiority of bER over ΔPCO_2_ in correlating with CHF is likely to be attributed partly to the ratio format of bER enabling a reduction of ventilatory fluctuations common to ΔPO_2_ and ΔPCO_2_, and partly to the physiological role of bER which takes into account the interaction between ΔPO_2_ and ΔPCO_2_. Besides, a regression model of ΔCBFv or ΔBOLD vs RGE metrics, including both ΔPCO_2_ and ΔPO_2_ as regressors would suffer from collinearity due to the correlation between ΔPCO_2_ and ΔPO_2_.

Interestingly, the spread of correlation strengths between ΔPO_2_ and ΔPCO_2_ as well as those between bER and ΔPO_2_ were different between TCD and MRI sessions (Fig 1). Correlation strengths might change in response to different factors which could include the postures of sitting up vs lying down, the noises, or the level of stress from the surroundings. Participants were sitting in an open and quiet environment in TCD sessions while they were lying down in supine position in a noisy environment of the MRI scanner bore. A change from the supine posture to sitting upright was reported to be associated with a redistribution of both blood flow and ventilation in the lungs, which affected the arterial PO_2_ [50–53]. While it is interesting to observe the differences in the correlation strength among RGE metrics between TCD and MRI sessions, the details of such mechanisms are outside the scope of our current study.

### Dynamic coupling between CHF and RGE metrics of bER, ΔPO_2_ and ΔPCO_2_

Correlation and coherence analyses showed the dynamic coupling between CHF (ΔCBFv and ΔBOLD) and RGE metrics of bER, ΔPO_2_ and ΔPCO_2_ in both TCD (Figs 2 and 3) and MRI sessions (Figs 3 and 4). Had ΔPO_2_ and ΔPCO_2_ been redundant, their correlation and coherence with CHF would be expected to be the same, and there should be weak correlation and coherence of CHF with bER. In contrast, comparing with ΔPO_2_ and bER, we found that ΔPCO_2_ had moderate to weak correlation with ΔCBFv (Fig 2). The map of β_ΔPCO2_ for a single individual showed fewer pixels with a significant association between ΔBOLD and ΔPCO_2_ (S4A Fig). Group analysis of regional β_ΔPCO2_ showed however almost a “null” map as only a few brain regions (insula, medial orbitofrontal and temporal areas) showed association between ΔPCO_2_ and ΔBOLD (S4B Fig) but such association did not reach statistical significance after correcting for multiple comparisons (Fig 4). Our group averaged result was consistent with the findings in the study by Golestani et al. [16], where large inter-individual variability was demonstrated in the brain maps of regressing BOLD signal changes on P_ET_CO_2_ during spontaneous breathing.

The interaction between ΔPCO_2_ and CHF is accompanied by an interaction between ΔPO_2_ and CHF. However, the mechanisms behind the superiority of ΔPO_2_ over ΔPCO_2_ in association with CHF, especially at the frequency range of 0.008–0.03Hz, remain unclear. A possible factor may be related to the difference in the O_2_ and CO_2_ dissociation curves, which are associated with the magnitude of the PO_2_ and PCO_2_ variations in time [13]. During spontaneous breathing, the PO_2_ varies from approximately 150mmHg in inspired air to 100mmHg in expired air at the top horizontal asymptote of the sigmoidal O_2_ dissociation curve, where it is associated with only a small change in oxygen saturation. On the contrary, the CO_2_ dissociation curve is more linear. With the Haldane effect, O_2_ affects the affinity of hemoglobin for CO_2_. Given the small fluctuations of oxygen saturation, a rather significant change in PO_2_ can, therefore, be associated with a minor change in PCO_2_. The larger oscillation amplitude of ΔPO_2_ in comparison with that of ΔPCO_2_ was observed by our team (Fig 2 and Table 2) and by Lenfant et al. [13]. An additional factor that was suggested by Lenfant et al. is the variation in the distribution of the ventilation-to-perfusion ratio (V/Q) in the lungs. Lenfant et al. reported that the alveolar-arterial difference in PO_2_ decreased as V/Q increased, while that in PCO_2_ remained nearly constant and independent of the changes in V/Q. The temporal oscillation of the V/Q distribution could then cause a more considerable change in PO_2_ than in PCO_2_. That also raises an intriguing idea for future research to examine any possible relationship between CHF and fluctuations in V/Q. The superiority of ΔPO_2_ over ΔPCO_2_ in association with CHF suggests a potentially important implication that CHF is more sensitive to the demand of the supply of O_2_ than of the removal of CO_2_. In addition to our studying of healthy subjects, exploring the interaction between CHF and RGE metrics in patients with known disorders may increase the understanding of the stronger coupling of CHF with bER and ΔPO_2_ than with ΔPCO_2_.

### Brain regions with the strongest bER-CHF coupling overlapped with many regions of default mode network

We used the resting state connectivity method with the seed at the left precuneus to outline the areas within DMN (Fig 5B). We found that brain regions with the strongest association between bER and ΔBOLD (Fig 5A) overlapped with many areas of DMN. Such strong association may be attributed to DMN showing higher metabolic and hemodynamic activities at rest than other parts of the cortex [23]. Fluctuations of systemic gases can be under the influences of ambient (exogenous) gases and/or systemic (endogenous) gases due to metabolism as well as feedbacks between chemoreceptors. Since the brain is one of the major shareholders of systemic blood flow [54] and metabolism [55, 56] at rest, any mechanisms that help to preserve cerebral metabolism and CBF within the normal range in the homeostatic regulatory process [12] may leave a signature in the CHF.

In our resting state experiments, CHF were measured on participating individuals during spontaneous breathing. No cognitive, motor, sensory, visual and gas challenges were applied to the participants. The internal environment of the body was steady, and major body organs had constant autonomic communication with the brain back and forth. At rest, background cerebral neurovascular coupling activities are characterized by DMN [23], and RGE metrics can be related to basal activities of the whole body. Hence it would be reasonable for RGE metrics to couple better with DMN than with other brain networks. bER which showed the strongest correlation with CHF is therefore expected to interact more with DMN. The bER-CHF coupling in DMN found in our study is consistent with the findings in previous studies that CBF and DMN connectivity were altered in individuals with disrupted systemic metabolism [57–64].

### Physiological processes that may be associated with the coherence between CHF and bER at the low frequency range of 0.008–0.03Hz

The CHF coherence with RGE metrics at the low frequency range of 0.008–0.03Hz may be associated with low frequency physiological processes in the brain that are grouped in B-wave frequency bandwidth. B-waves with a period of 0.5 to 2 minutes (0.008–0.03Hz) have been reported to be related to autoregulation of microvasculature, spontaneous rhythmic oscillations in intracranial pressure (ICP), and intrinsic brainstem rhythm that leads to cerebral blood volume modulation [65, 66]. B-waves may also independently reflect the neurovascular coupling process by altering vascular diameters to ensure the delivery of O_2_ and other circulating metabolites through the contractile properties of pericytes or vascular smooth muscle cells [67–70]. Since oscillations of B-waves are not always associated with the change of arterial CO_2_ [66], it would be interesting to pursue further the relationship between B-waves and all three of our RGE metrics as well as resting state CHF.

The coherence of CHF with RGE metrics at the frequency of 0.008–0.03Hz may also be related to a proposed mechanism to remove cerebral metabolic waste. In the glymphatic model [71], cerebral metabolic waste was hypothesized to be cleared from the brain via the perivascular space by vascular pulsation. The actual type of vascular pulsation remains to be determined. To describe possible mechanisms for glymphatic convection, Kiviniemi et al. [72] identified a very slow pulsation found in cerebrospinal fluid (0.001–0.023Hz), which is again in the frequency range similar to the oscillations of the RGE metrics.

In the peripheral circulation, laser Doppler measurements showed that the endothelial activity oscillated at the frequency of 0.0096–0.021Hz [73, 74], which is within the same low frequency range of CHF and fluctuations of RGE metrics with a particular interest on bER based on our results. Is there any direct relationship between oscillatory cycles of peripheral endothelial activity and CHF? Considering the model of diving reflex [75] where an increase in CBF is associated with peripheral vasoconstriction, is it possible that the decrease in peripheral blood flow is mirrored by an increase in CBF in a homeostatic process during spontaneous breathing? Future research can be directed to explore the association between bER, CHF and peripheral (skin) blood flow oscillations at the low frequency range of 0.008–0.03 Hz.

Separate from the B-waves and glymphatic model, we do not consider heart rate (HR) or heart rate variability (HRV) to be a major contributing factor for the role of RGE metrics in CHF. Even though HR and HRV had been reported to contribute to low frequency fluctuations, the peaks of coherence between ΔBOLD and HR/HRV were at the frequency of 0.05Hz or above (period ~30–42 seconds) [5, 76] which are different from our findings that RGE metrics were coherent with CHF between 0.008 and 0.03Hz.

### Ventilatory volume fluctuations are not the primary origin of our finding on the association between CHF and the fluctuations of RGE metrics

We should point out that ventilatory volume fluctuations are unlikely to be the cause of our observed interaction between CHF and RGE metrics. Respiratory variability, namely changes in respiration volume per time or RVT that had been shown to have a strong correlation with the change in respiratory volume [14] had also been discussed as a possible source for DMN activities [3]. However, a number of considerations showed that RVT did not provide the answers to our findings on the differences observed between ΔPO_2_ and ΔPCO_2_ in their interaction with CHF. First, RVT would be expected to have the same effect on the time courses of ΔPO_2_ and ΔPCO_2_. Second, bER, being a ratio of ΔPO_2_/ΔPCO_2_, reduces the contribution of ventilatory volume fluctuations to the interaction between bER and CHF. Previous studies showed that the relationship between the time courses of P_ET_CO_2_ and RVT was unclear [14, 16]. There was only a mild coherence between P_ET_CO_2_ and RVT [14]. The authors in these studies showed that changes in RVT had a weaker correlation with BOLD signal changes when compared with P_ET_CO_2_ [16, 77]. Such results are not surprising as P_ET_CO_2_ is supposed to play a more direct role than ventilation to modulate CHF [3, 4]. Besides, the time course of RVT is less than ideal as the numerator of RVT acquired with a respiratory bellow indicates the chest excursion, while respiratory volume is the volume of air breathing in or out. Our coherence analysis showed that ΔBOLD at DMN regions were less coherent with RVT than with bER or ΔPO_2_ (S5 Fig). RVT showed coherence with ΔBOLD at the phase lag of 0±π/2 between 0.016 and 0.031Hz but the coherence increased slightly between 0.008 and 0.016Hz at the phase lag of π±π/2. The coherence findings of RVT were also different from those of ΔPCO_2_. The frequency bandwidths and the phase differences seen in our coherence of RVT with ΔBOLD are consistent with those reported by Van den Aardweg [14] even though his team was measuring respiratory volume from a facemask. While coherence frequency bandwidths at different phase lags may be referring to the responses from different chemoreceptors (e.g. central vs peripheral), the relationship between the time courses of P_ET_CO_2_ and RVT is still unclear.

### Technical issues that have been addressed

In this study, we used TCD to acquire CHF in addition to using fMRI. One advantage of TCD is its high temporal resolution with less concern on the aliasing effect of high frequency hemodynamic signal. Potential technical confounds like gas sampling at two gas analyzers and technical delay on the time series were also addressed in the data acquisition protocol. In the gas sampling circuit, the same gas sample volume was used in both CO_2_ and O_2_ analyzers at the same gas sampling flow rate. The technical delay on the time series of gas measurements due to transit time of respiratory gases and the response of the equipment were corrected in the preprocessing of the physiological signals.

In the preprocessing of fMRI data, we did not apply RETROICOR [78] to reduce ventilatory and cardiac ‘interference’ in the BOLD signals because the heart rate was reported to contribute to BOLD signals mainly at 0.3Hz or above [76] which was above the frequency bandwidths of interest (below 0.03Hz) in this study. Our findings of the correlation between ΔBOLD and RGE metrics in healthy subjects were supported by the CBFv data which were acquired with TCD at a sampling rate of 100Hz on the same group of subjects. There is no concern of aliasing of high frequency cardiac signal into our CBFv signal at low frequency range below 0.03Hz. In Fig 2, we also demonstrated that the preprocessing step for CBFv signals like data smoothing did not change the correlation ranking of ΔCBFv with bER, ΔPO_2_ and ΔPCO_2_.

In our fMRI regression analysis, we did not convolve the RGE metrics with a specific respiratory response function (RRF) which was commonly used to convolve with RVT or P_ET_CO_2_ to improve its correlation with ΔBOLD [3, 4, 6, 16]. The reason is that RRF is unnecessary when our primary objective is comparing the association of different RGE metrics (bER, ΔPO_2_ and ΔPCO_2_) with ΔBOLD. If the application of RRF improves the correlation of ΔPCO_2_ with ΔBOLD, it will also improve the correlation of ΔPO_2_ or bER with ΔBOLD. There is no justification for applying RRF to only ΔPCO_2_ and not to ΔPO_2_. Applying the same RRF to all of the RGE metrics should have little effect on how one RGE metric ranks against other RGE metrics in its association with ΔBOLD. Another approach to state that convolution of RGE metrics with RRF is not applicable in our case because our research question is ‘Which RGE metric contributes more to ΔBOLD?’ whereas studies that took up RRF [3, 4, 6, 16] focused on a separate question of ‘Which brain regions have stronger ΔBOLD responses to RVT or P_ET_CO_2_?’ With a different research question, it was reasonable for those previous studies to focus on convolving the RVT or P_ET_CO_2_ time series with RRF which identified the portion of the ΔBOLD that was not directly accounted for by the time series of RVT or P_ET_CO_2_.

### Technical issues that need to be addressed in the future

Even though ΔPO_2_ is related to the partial pressure of O_2_ utilized in the whole body, we cannot simply substitute ΔPO_2_ and ΔPCO_2_ for O_2_ uptake (VO_2_) and CO_2_ release (VCO_2_). O_2_ uptake and CO_2_ release require extra elements like respiratory minute volume (sometimes normalized for body weight) and are typically evaluated as a steady state of RGE data obtained by averaging the breath-by-breath signal over minutes. A measurement of breath-by-breath VO_2_ and VCO_2_ inside the MRI scanner in addition to the collection of ΔPO_2_ and ΔPCO_2_ was not included in our study. Given the physical constraint of the MRI settings, we collected ΔPO_2_ and ΔPCO_2_ using a nasal tubing. We will leave the possibility of measuring VO_2_ and VCO_2_ during MRI to future neuroimaging research. However, under specific conditions, some information extracted from ΔPO_2_ and ΔPCO_2_ can be related to that from VO_2_ and VCO_2_. As mentioned above, bER has the explicit influence on suppressing the ventilatory volume when we are taking the ratio of ΔPO_2_ and ΔPCO_2_. We, therefore, consider the possibility of bER or equivalently ΔPO_2_/ΔPCO_2_ being a surrogate for breath-by-breath VO_2_/VCO_2_ at rest.

As the vasodilatory role of CO_2_ to modulate CBF is an entrenched belief, it is natural to gravitate towards the explanation that association between RGE metrics and CHF must be resulting from “the effect of CO_2_ which reflects in O_2_”. However, the model of CO_2_ being the sole agent to interact with CHF remains at variance with our findings of non-redundancy between ΔPO_2_ and ΔPCO_2_ in this study. Therefore, further investigations on the roles of bER, ΔPO_2_ and ΔPCO_2_ in their association with CHF would be warranted.

### Potential application of bER in the evaluation of CVR

While bER was prominently coupled with CHF within DMN, bER was also coupled, albeit to a less extent, with CHF in the rest of voxels throughout the whole brain. Therefore bER can be used instead of ΔPCO_2_ as a regressor to evaluate CVR to spontaneous breathing (S6 Fig). The question is whether the expected small perturbations provided by RGE metrics during spontaneous breathing could have any clinical utility. The previous study on the successful identification of local vascular deficits of moyamoya patients [79] had already been reported for CVR to P_ET_CO_2_ obtained in spontaneous breathing. In our recent study, we found that bER is more robust than P_ET_CO_2_ in characterizing the cerebral hemodynamic responses to brief breath hold challenge in healthy adults [15]. Future studies will clarify the sensitivity and benefits of imaging local vascular deficits using bER instead of P_ET_CO_2_ from spontaneous breathing.

Imaging of bER-CHF coupling may open up opportunities for potential clinical diagnosis and intervention. The clinical potential of RER at rest has been reported for decades. RER, the reciprocal of the steady state mean value of bER, has been reported to change significantly with age [80] and with diseases attributed to respiratory [81], liver [63], cardiac [82] and neuronal [83] dysfunctions. Instead of focusing on the steady state results, our manuscript centers more on the dynamic fluctuations of bER, as a separate metric of interest. The bER-CHF coupling may offer an alternative approach to map brain regions interacting with systemic homeostatic processes besides resting state connectivity analysis for the connections among brain regions [2] and task-induced negative BOLD responses [23]. Since abnormal DMN can be an indicator of neuronal disorders [84, 85], future studies on different patient populations may help clarify the meaning of abnormal bER-CHF coupling. Our brain-body coupling framework may be applied in the investigation of brain responses to voluntary respiratory techniques like meditation, yoga breathing or tai-chi [86–93] to manipulate RGE for intervention purpose. Instrument assisted respiratory techniques like the Bi-level Positive Airway Pressure (BiPAP) [94–96] that applies to sleep apnea or Amyotrophic Lateral Sclerosis (ALS) may also be considered.

## Conclusion

While the physiological mechanisms are not completely known for the interaction between CHF and RGE metrics of bER, ΔPO_2_ and ΔPCO_2_, our findings provide evidence that fluctuations of RGE metrics are associated with resting state CHF at a low frequency between 0.008Hz and 0.03 Hz. Both bER and ΔPO_2_ are more superior to ΔPCO_2_ in association with CHF while CHF could correlate more strongly with bER than with ΔPO_2_ in some brain regions. Brain regions with the strongest bER-CHF coupling overlap with many areas of DMN. In addition to offering a physiological model to characterize the contribution of gas exchange elements in the low frequency resting state fluctuations, our findings provide new directions to the study of brain-body interaction. Assessing the validity of the popular CO_2_-only hypothesis as well as other alternative hypotheses to explain our results of bER-CHF coupling remains an on-going project.

## Supporting information

S1 Fig Definition of end inspiration and end expiration on the time series of RGE metrics. A segment of 90-second time series of ΔCBFv in left MCA and physiological changes including breath-by-breath bER, ΔPO_2_, ΔPCO_2_, P_ET_O_2_ and P_ET_CO_2_ measured by gas analyzers and respiration time series (Resp) measured by respiratory bellow in a representative subject during spontaneous breathing in TCD session. Open circles represent end expiration while closed circles represent end inspiration. Positive phases with deflection above zero on the respiration time series represent inspiration and negative phases with deflection below zero represent expiration. The inspiratory and expiratory phases of each respiratory cycle on the time series of P_ET_O_2_ and P_ET_CO_2_ are verified by those on respiration time series. The timing for open (end expiration) and closed (end inspiration) circles in green is the same as those in red and blue.
(TIF)

S2 Fig Correlation of ΔCBFv with the ratio as well as the product of ΔPO_2_ and ΔPCO_2_ in a representative subject. Time series of ΔCBFv and bER normalized to its mean (*upper left*). Moderate correlation was shown between ΔCBFv and bER (*upper right*). Time series of ΔCBFv and the product of ΔPO_2_ and ΔPCO_2_ (ΔPO_2_×ΔPCO_2_) normalized to its mean (*lower left*). Weak correlation was shown between ΔCBFv and ΔPO_2_×ΔPCO_2_ (*lower right*).
(TIF)

S3 Fig Wavelet transform coherence analysis between bER and ΔCBFv in a representative subject. (A) Time series of bER and ΔCBFv measured in right MCA of a representative subject at rest. (B) The squared wavelet coherence between these two time series. Squared wavelet coherence is plotted with x-axis as time and y-axis as scale which has been converted to its equivalent Fourier period. The magnitude of wavelet transform coherence ranges between 0 and 1, where warmer color represents stronger coherence and cooler color represents weaker coherence. Areas inside the ‘cone of influence’, which are locations in the time-frequency plane where edge effects give rise to lower confidence in the computed values, are shown in faded color outside of the conical contour. The statistical significance level of the wavelet coherence is estimated using Monte Carlo methods and the 5% significance level against red noise is shown as thick contour. The phase angle between the two time series, with bER leading ΔCBFv, at particular samples of the time-frequency plane is indicated by an arrow (rightward pointing arrows indicate that the time series are in phase or positively correlation, leftward pointing arrows indicate anticorrelation and the downward pointing arrows indicate phase angles of π/2). There are four different ranges of phase lags: 0+π/2, 0-π/2, π-π/2, and π+π/2. (C) Time-averaged coherence at four different phase lags of 0+π/2, 0-π/2, π-π/2, and π+π/2. At each phase lag range, time-averaged coherence was defined as the total significant coherence at each scale where the wavelet coherence magnitude exceeded 95% significance level, normalized by the maximum possible coherence outside the cone of influence, i.e. inside the conical contour, at that particular scale and phase lag range.
(TIF)

S4 Fig Regional association between ΔBOLD and RGE metrics in a representative subject and in group. (A) Brain maps of β_bER_, β_ΔPO2_ and β_ΔPCO2_ in a representative subject after correcting for multiple comparisons. (B) Group maps of regional β_bER_, β_ΔPO2_ and β_ΔPCO2_ before correcting for multiple comparisons for all the subjects included in the MRI sessions.
(TIF)

S5 Fig Distribution of time-averaged coherence between time series of respiratory volume per unit time (RVT) and ΔBOLD in brain regions within the DMN. The mean time-averaged coherence between time series of RVT and ΔBOLD at the phase lags of 0±π/2 and π±π/2 (thick color lines) in the brain regions of the inferior parietal lobule (IPL), posterior cingulate (PCC) and precuneus (PCun) of the left brain (*left panel*) and of the right brain (*right panel*) (n = 10). Color shaded areas represent standard error of the mean. Coherence between two time series at the phase lag of 0±π/2 indicates positive correlation, while negative correlation is represented by the coherence at the phase lag of π±π/2.
(TIF)

S6 Fig Regional CVR maps under exogenous CO2 challenge and regional association between ΔBOLD and RGE metrics during spontaneous breathing. The CVR map during spontaneous breathing indicated by β_bER_ changes resembled the CVR map under exogenous CO_2_ challenge indicated by CVR_CO2-PETCO2_.
(TIF)

S1 Table Correlation among RGE metrics. Strength of correlation indicated by Pearson’s correlation coefficients among respiratory metrics including bER, ΔPO_2_ and ΔPCO_2_ in all subjects who participated in TCD sessions (n = 13), and those who participated in MRI sessions (n = 20).
(DOCX)

S2 Table Correlation between RGE metrics and ΔCBFv in TCD sessions. Strength of correlation indicated by Pearson’s correlation coefficients between ΔCBFv and RGE metrics including bER, ΔPO_2_, ΔPCO_2_ and P_ET_CO_2_ (n = 13). Numbers in brackets next to Pearson’s correlation coefficients indicate p values from individual correlation analyses. The bottom row shows the mean values of Fisher’s Z scores transformed from Pearson’s correlation coefficients in groups. Numbers in brackets next to mean Fisher’s Z scores indicate p values in the paired comparisons.
(DOCX)
