# Supplementary figures and images for: Dynamic brain-body coupling of breath-by-breath O2-CO2 exchange ratio with resting state cerebral hemodynamic fluctuations
Source: PLoS One. 2020 Sep 21;15(9):e0238946. doi: 10.1371/journal.pone.0238946 (PMC7505589; doi:10.1371/journal.pone.0238946)

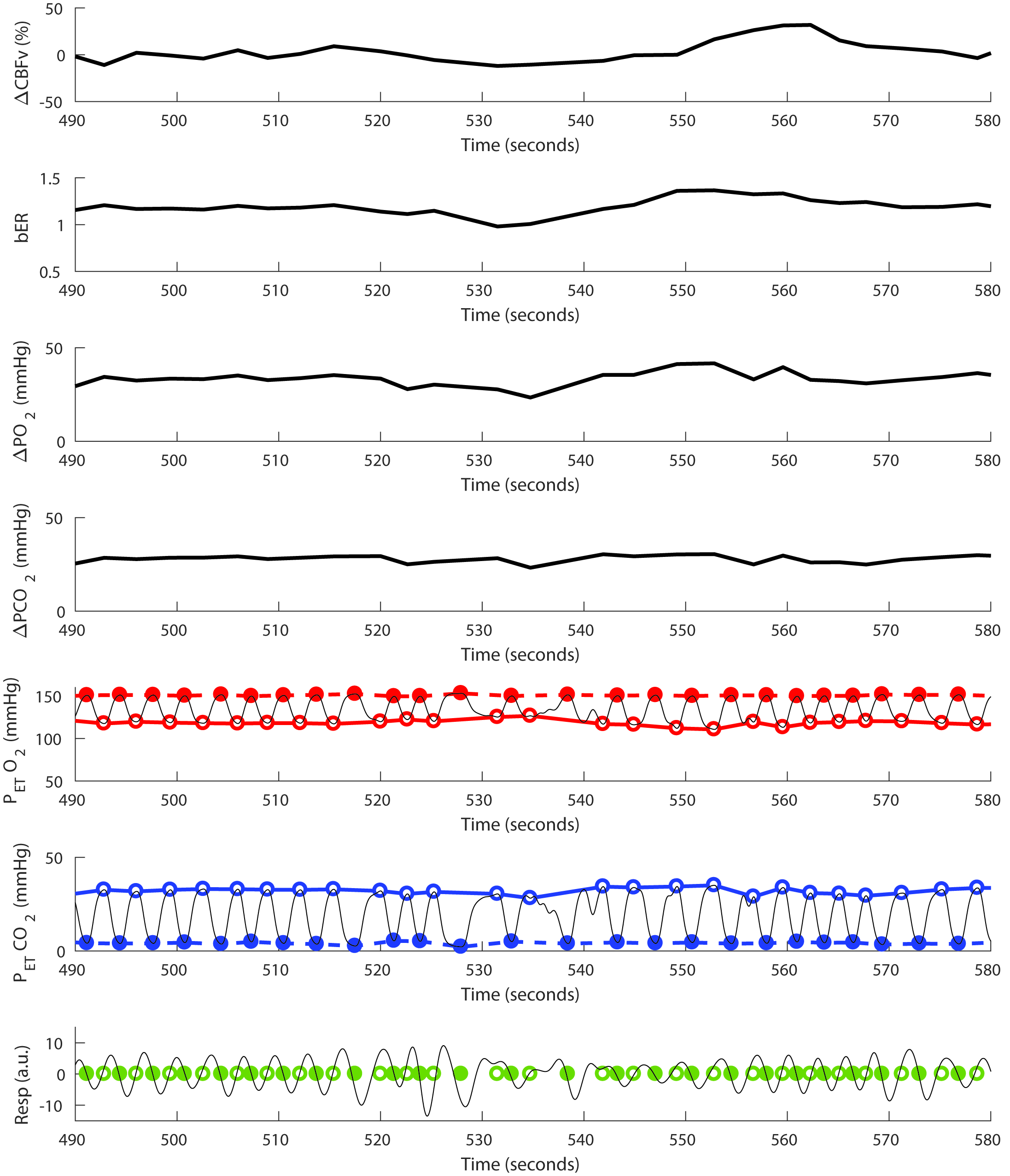

Supplement: S1 Fig — A segment of 90-second time series of ΔCBFv in left MCA and physiological changes including breath-by-breath bER, ΔPO2, ΔPCO2, PETO2 and PETCO2 measured by gas analyzers and respiration time series (Resp) measured by respiratory bellow in a representative subject during spontaneous breathing in TCD session. Open circles represent end expiration while closed circles represent end inspiration. Positive phases with deflection above zero on the respiration time series represent inspiration and negative phases with deflection below zero represent expiration. The inspiratory and expiratory phases of each respiratory cycle on the time series of PETO2 and PETCO2 are verified by those on respiration time series. The timing for open (end expiration) and closed (end inspiration) circles in green is the same as those in red and blue. (TIF) [file pone.0238946.s001.tif]

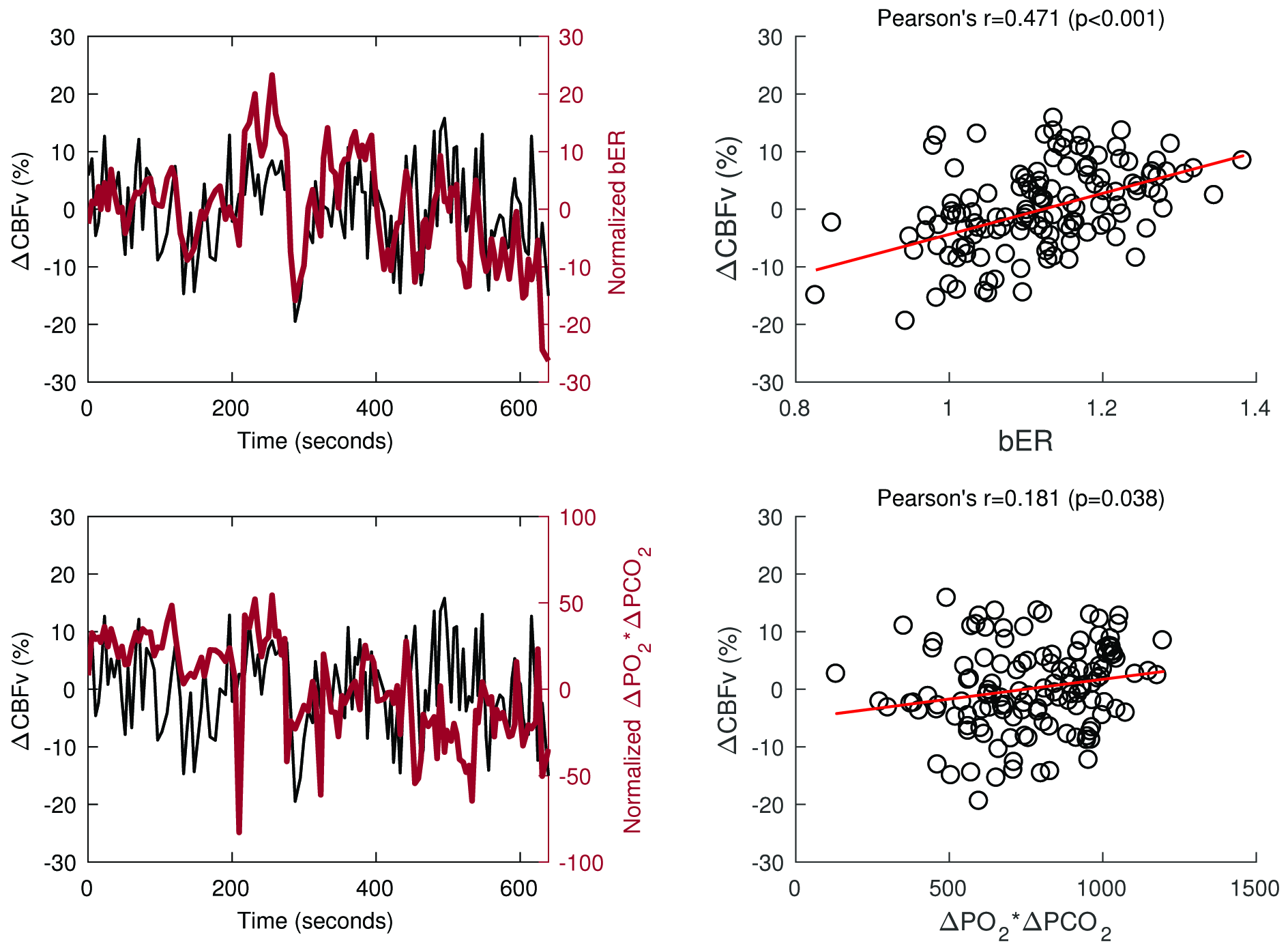

Supplement: S2 Fig — Time series of ΔCBFv and bER normalized to its mean (upper left). Moderate correlation was shown between ΔCBFv and bER (upper right). Time series of ΔCBFv and the product of ΔPO2 and ΔPCO2 (ΔPO2×ΔPCO2) normalized to its mean (lower left). Weak correlation was shown between ΔCBFv and ΔPO2×ΔPCO2 (lower right). (TIF) [file pone.0238946.s002.tif]

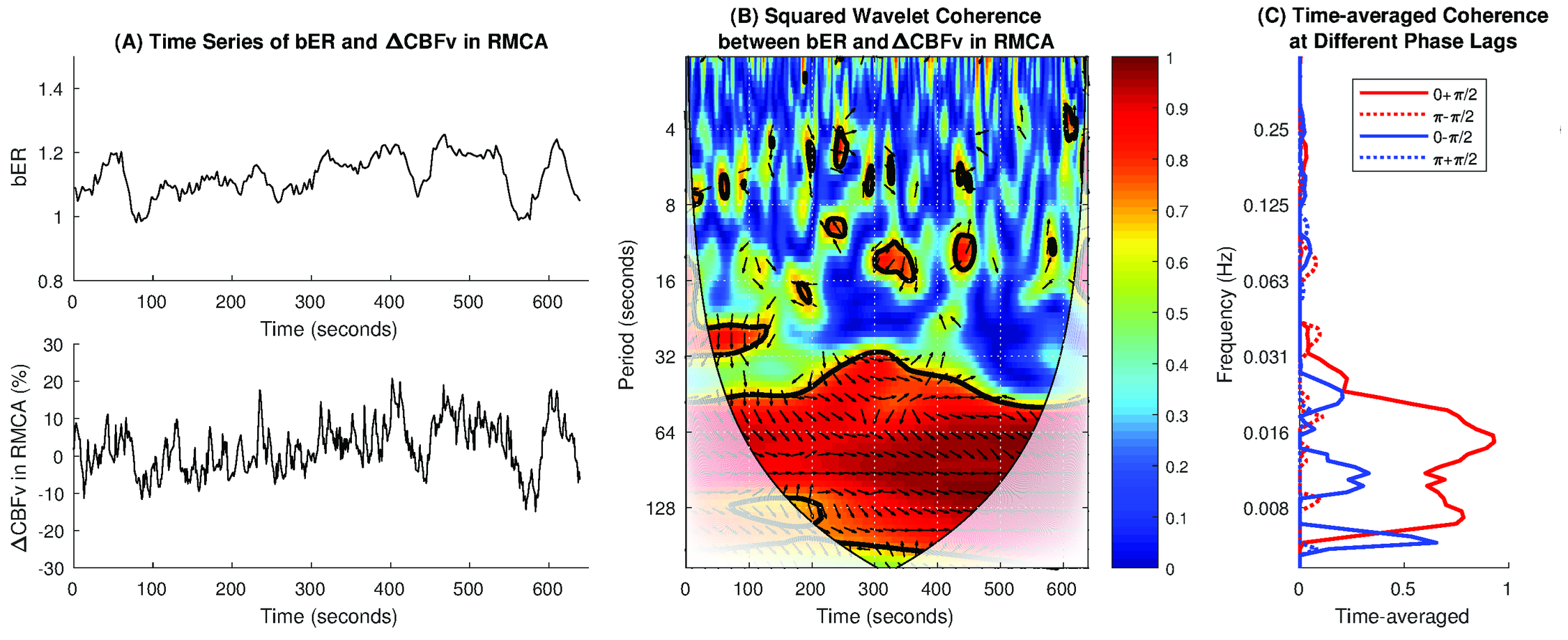

Supplement: S3 Fig — (A) Time series of bER and ΔCBFv measured in right MCA of a representative subject at rest. (B) The squared wavelet coherence between these two time series. Squared wavelet coherence is plotted with x-axis as time and y-axis as scale which has been converted to its equivalent Fourier period. The magnitude of wavelet transform coherence ranges between 0 and 1, where warmer color represents stronger coherence and cooler color represents weaker coherence. Areas inside the ‘cone of influence’, which are locations in the time-frequency plane where edge effects give rise to lower confidence in the computed values, are shown in faded color outside of the conical contour. The statistical significance level of the wavelet coherence is estimated using Monte Carlo methods and the 5% significance level against red noise is shown as thick contour. The phase angle between the two time series, with bER leading ΔCBFv, at particular samples of the time-frequency plane is indicated by an arrow (rightward pointing arrows indicate that the time series are in phase or positively correlation, leftward pointing arrows indicate anticorrelation and the downward pointing arrows indicate phase angles of π/2). There are four different ranges of phase lags: 0+π/2, 0-π/2, π-π/2, and π+π/2. (C) Time-averaged coherence at four different phase lags of 0+π/2, 0-π/2, π-π/2, and π+π/2. At each phase lag range, time-averaged coherence was defined as the total significant coherence at each scale where the wavelet coherence magnitude exceeded 95% significance level, normalized by the maximum possible coherence outside the cone of influence, i.e. inside the conical contour, at that particular scale and phase lag range. (TIF) [file pone.0238946.s003.tif]

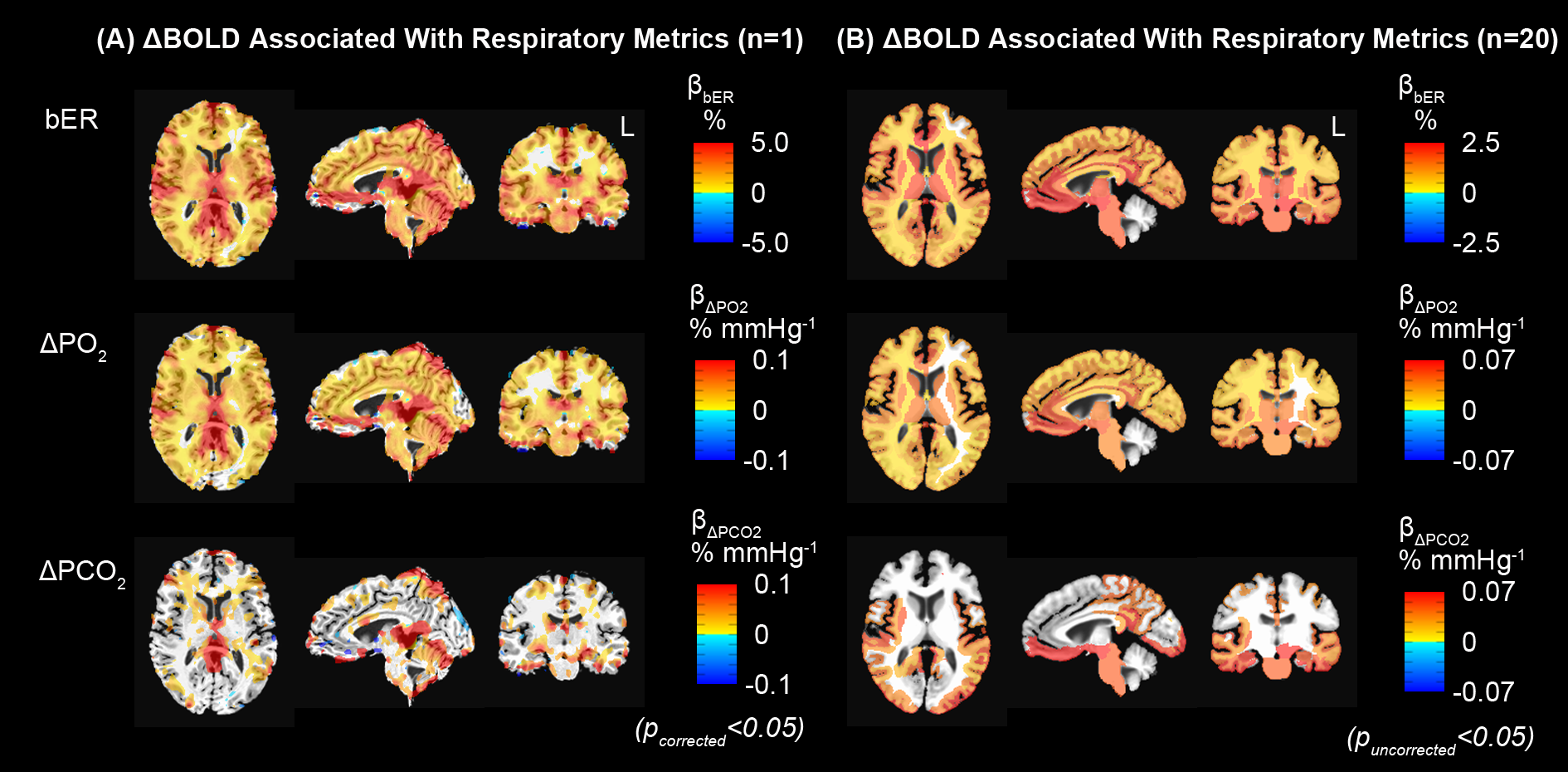

Supplement: S4 Fig — (A) Brain maps of βbER, βΔPO2 and βΔPCO2 in a representative subject after correcting for multiple comparisons. (B) Group maps of regional βbER, βΔPO2 and βΔPCO2 before correcting for multiple comparisons for all the subjects included in the MRI sessions. (TIF) [file pone.0238946.s004.tif]

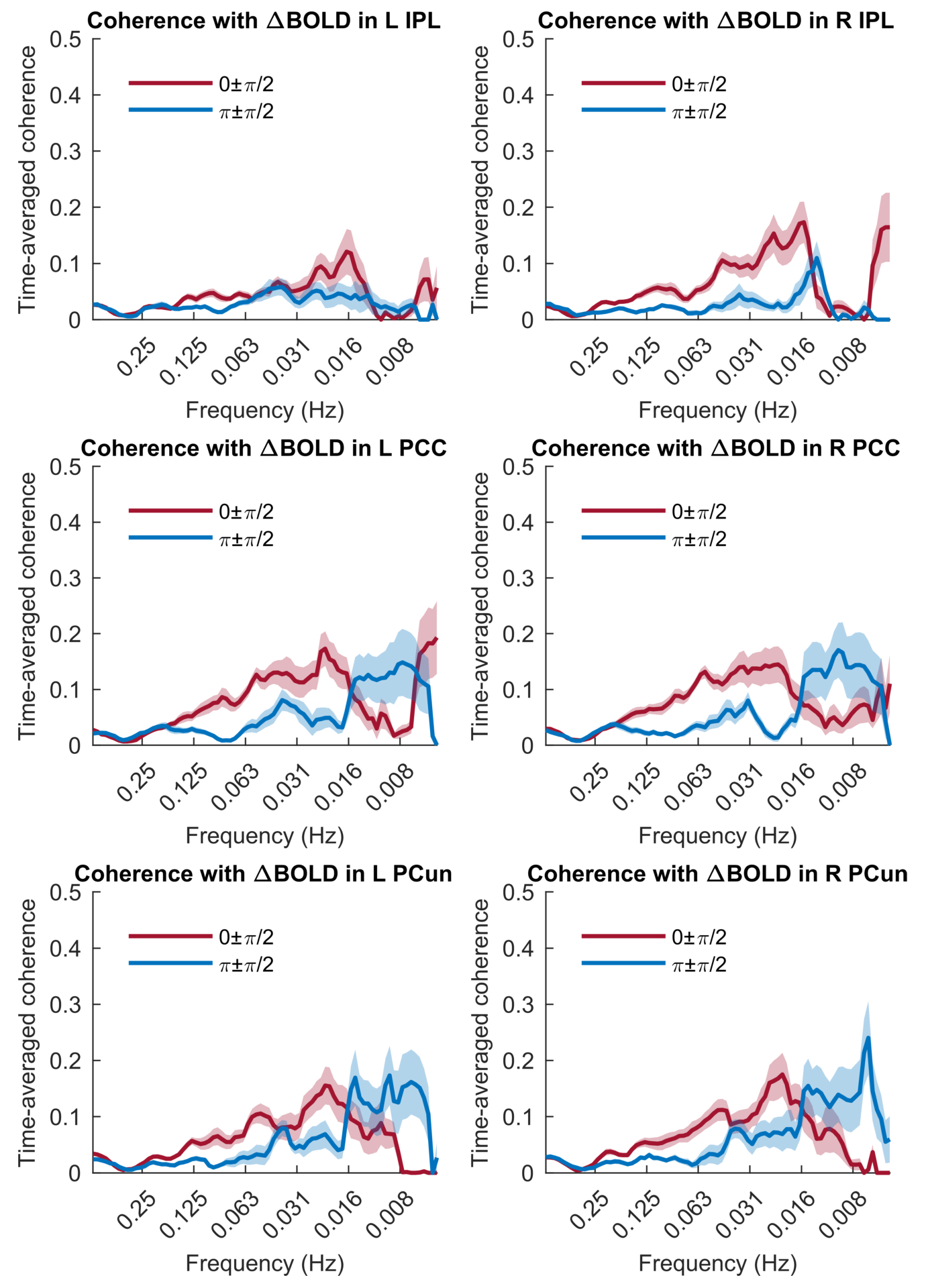

Supplement: S5 Fig — The mean time-averaged coherence between time series of RVT and ΔBOLD at the phase lags of 0±π/2 and π±π/2 (thick color lines) in the brain regions of the inferior parietal lobule (IPL), posterior cingulate (PCC) and precuneus (PCun) of the left brain (left panel) and of the right brain (right panel) (n = 10). Color shaded areas represent standard error of the mean. Coherence between two time series at the phase lag of 0±π/2 indicates positive correlation, while negative correlation is represented by the coherence at the phase lag of π±π/2. (TIF) [file pone.0238946.s005.tif]

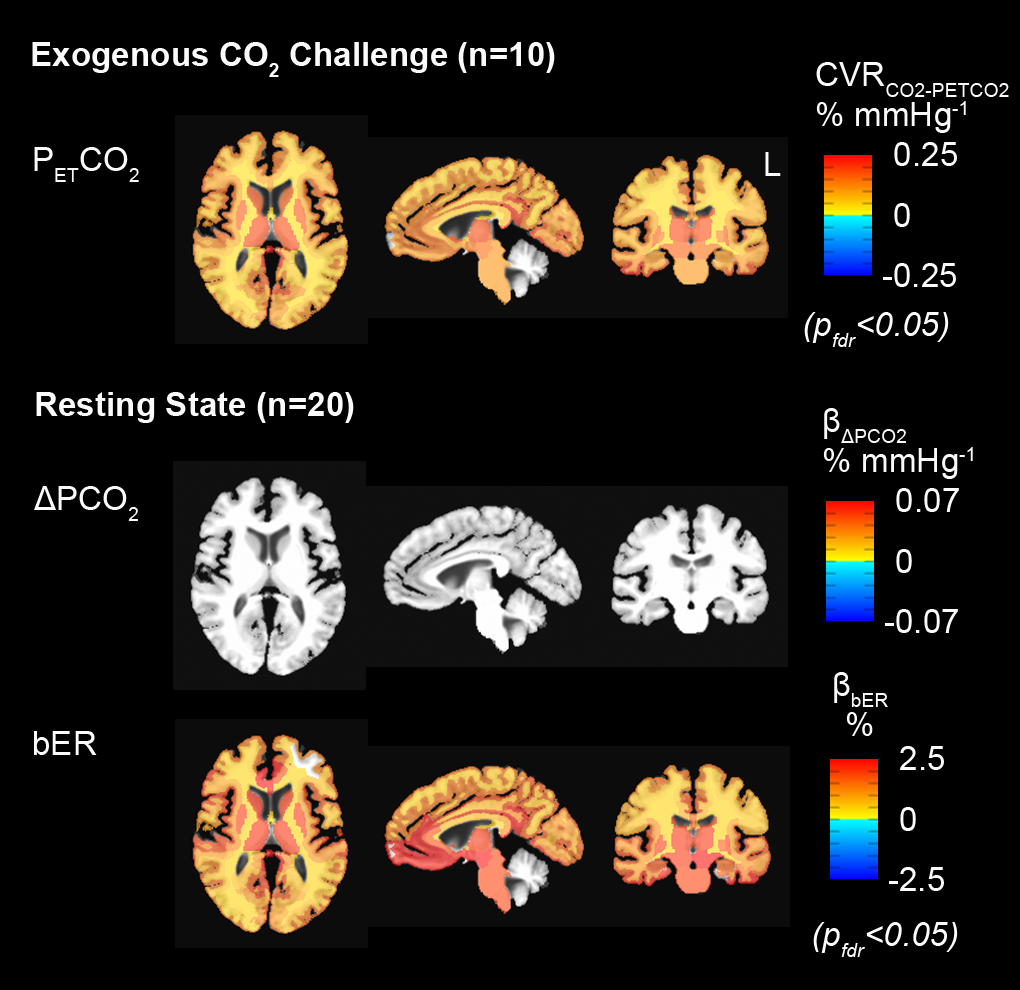

Supplement: S6 Fig — The CVR map during spontaneous breathing indicated by βbER changes resembled the CVR map under exogenous CO2 challenge indicated by CVRCO2-PETCO2. (TIF) [file pone.0238946.s006.tif]
