# Supplementary material for: Dynamic brain-body coupling of breath-by-breath O2-CO2 exchange ratio with resting state cerebral hemodynamic fluctuations
Source: PLoS One. 2020 Sep 21;15(9):e0238946. doi: 10.1371/journal.pone.0238946 (PMC7505589; doi:10.1371/journal.pone.0238946)
Supplement: S1 Table — Strength of correlation indicated by Pearson’s correlation coefficients among respiratory metrics including bER, ΔPO2 and ΔPCO2 in all subjects who participated in TCD sessions (n = 13), and those who participated in MRI sessions (n = 20). (DOCX) [file pone.0238946.s007.docx]

**S1 Table. Correlation among RGE metrics.**

|  | **TCD** | | | | **BOLD** | | |
| --- | --- | --- | --- | --- | --- | --- | --- |
| **Subjects** | **ΔPO_2_ vs ΔPCO_2_** | **bER vs ΔPO_2_** | **bER vs ΔPCO_2_** | | **ΔPO_2_ vs ΔPCO_2_** | **bER vs ΔPO_2_** | **bER vs ΔPCO_2_** |
| s1 | --- | --- | | --- | 0.672 (<0.001) | 0.895 (<0.001) | 0.272 (<0.001) |
| s2 | --- | --- | | --- | 0.437 (<0.001) | 0.280 (0.002) | -0.740 (<0.001) |
| s3 | --- | --- | | --- | 0.191 (0.085) | 0.379 (<0.001) | -0.830 (<0.001) |
| s4 | --- | --- | | --- | 0.774 (<0.001) | 0.860 (<0.001) | 0.343 (<0.001) |
| s5 | --- | --- | | --- | 0.667 (<0.001) | 0.678 (<0.001) | -0.043 (0.556) |
| s6 | --- | --- | | --- | 0.896 (<0.001) | 0.901 (<0.001) | 0.633 (<0.001) |
| s7 | --- | --- | | --- | 0.956 (<0.001) | 0.994 (<0.001) | 0.921 (<0.001) |
| s8 | --- | --- | | --- | 0.778 (<0.001) | 0.728 (<0.001) | 0.137 (0.100) |
| s9 | --- | --- | | --- | 0.419 (<0.001) | 0.764 (<0.001) | -0.261 (0.003) |
| s10 | 0.844 (<0.001) | 0.934 (<0.001) | | 0.598 (<0.001) | 0.814 (<0.001) | 0.907 (<0.001) | 0.495 (<0.001) |
| s11 | 0.807 (<0.001) | 0.726 (<0.001) | | 0.180 (0.026) | 0.804 (<0.001) | 0.869 (<0.001) | 0.419 (<0.001) |
| s12 | 0.938 (<0.001) | 0.969 (<0.001) | | 0.833 (<0.001) | 0.852 (<0.001) | 0.844 (<0.001) | 0.441 (<0.001) |
| s13 | 0.731 (<0.001) | 0.929 (<0.001) | | 0.427 (<0.001) | 0.846 (<0.001) | 0.985 (<0.001) | 0.740 (<0.001) |
| s14 | 0.825 (<0.001) | 0.846 (<0.001) | | 0.399 (<0.001) | 0.799 (<0.001) | 0.894 (<0.001) | 0.446 (<0.001) |
| s15 | 0.878 (<0.001) | 0.526 (<0.001) | | 0.057 (0.518) | 0.845 (<0.001) | 0.733 (<0.001) | 0.256 (0.001) |
| s16 | 0.879 (<0.001) | 0.736 (<0.001) | | 0.327 (<0.001) | 0.528 (<0.001) | 0.879 (<0.001) | 0.062 (0.416) |
| s17 | 0.809 (<0.001) | 0.937 (<0.001) | | 0.552 (<0.001) | 0.830 (<0.001) | 0.939 (<0.001) | 0.588 (<0.001) |
| s18 | 0.766 (<0.001) | 0.971 (<0.001) | | 0.593 (<0.001) | 0.798 (<0.001) | 0.972 (<0.001) | 0.644 (<0.001) |
| s19 | 0.891 (<0.001) | 0.960 (<0.001) | | 0.729 (<0.001) | 0.460 (<0.001) | 0.840 (<0.001) | -0.094 (0.217) |
| s20 | 0.752 (<0.001) | 0.572 (<0.001) | | -0.101 (0.188) | 0.785 (<0.001) | 0.849 (<0.001) | 0.342 (<0.001) |
| s21 | 0.932 (<0.001) | 0.750 (<0.001) | | 0.488 (<0.001) | --- | --- | --- |
| s22 | 0.895 (<0.001) | 0.752 (<0.001) | | 0.382 (<0.001) | --- | --- | --- |

Strength of correlation indicated by Pearson’s correlation coefficients among respiratory metrics including bER, ΔPO_2_ and ΔPCO_2_ in all subjects who participated in TCD sessions (n=13), and those who participated in MRI sessions (n=20).
