# Supplementary material for: Dynamic brain-body coupling of breath-by-breath O2-CO2 exchange ratio with resting state cerebral hemodynamic fluctuations
Source: PLoS One. 2020 Sep 21;15(9):e0238946. doi: 10.1371/journal.pone.0238946 (PMC7505589; doi:10.1371/journal.pone.0238946)
Supplement: S2 Table — Strength of correlation indicated by Pearson’s correlation coefficients between ΔCBFv and RGE metrics including bER, ΔPO2, ΔPCO2 and PETCO2 (n = 13). Numbers in brackets next to Pearson’s correlation coefficients indicate p values from individual correlation analyses. The bottom row shows the mean values of Fisher’s Z scores transformed from Pearson’s correlation coefficients in groups. Numbers in brackets next to mean Fisher’s Z scores indicate p values in the paired comparisons. (DOCX) [file pone.0238946.s008.docx]

**S2 Table. Correlation between RGE metrics and ∆CBFv in TCD sessions.**

|  | **ΔCBFv in LMCA** | | | | **ΔCBFv in RMCA** | | | |
| --- | --- | --- | --- | --- | --- | --- | --- | --- |
| **Subjects** | **bER** | **ΔPO_2_** | **ΔPCO_2_** | **P_ET_CO_2_** | **bER** | **ΔPO_2_** | **ΔPCO_2_** | **P_ET_CO_2_** |
| s10 | 0.400 (<0.001) | 0.423 (<0.001) | 0.367 (<0.001) | 0.368 (<0.001) | 0.678 (<0.001) | 0.628 (<0.001) | 0.394 (<0.001) | 0.399 (<0.001) |
| s11 | 0.525 (<0.001) | 0.406 (<0.001) | 0.126 (0.121) | 0.159 (0.051) | 0.207 (0.011) | 0.243 (0.003) | 0.167 (0.040) | 0.179 (0.027) |
| s12 | 0.858 (<0.001) | 0.849 (<0.001) | 0.752 (<0.001) | 0.776 (<0.001) | 0.877 (<0.001) | 0.854 (<0.001) | 0.736 (<0.001) | 0.763 (<0.001) |
| s13 | 0.505 (<0.001) | 0.400 (<0.001) | 0.052 (0.521) | 0.082 (0.313) | 0.379 (<0.001) | 0.380 (<0.001) | 0.231 (0.004) | 0.234 (0.004) |
| s14 | 0.459 (<0.001) | 0.464 (<0.001) | 0.313 (<0.001) | 0.370 (<0.001) | 0.362 (<0.001) | 0.289 (<0.001) | 0.121 (0.104) | 0.130 (0.080) |
| s15 | 0.295 (0.001) | 0.301 (<0.001) | 0.188 (0.032) | 0.193 (0.027) | 0.461 (<0.001) | 0.325 (<0.001) | 0.113 (0.199) | 0.124 (0.159) |
| s16 | 0.571 (<0.001) | 0.314 (<0.001) | 0.039 (0.656) | 0.032 (0.721) | 0.471 (<0.001) | 0.254 (0.003) | 0.019 (0.826) | 0.017 (0.850) |
| s17 | --- | --- | --- | --- | 0.293 (<0.001) | 0.356 (<0.001) | 0.359 (<0.001) | 0.359 (<0.001) |
| s18 | 0.780 (<0.001) | 0.762 (<0.001) | 0.475 (<0.001) | 0.483 (<0.001) | 0.758 (<0.001) | 0.755 (<0.001) | 0.503 (<0.001) | 0.501 (<0.001) |
| s19 | 0.723 (<0.001) | 0.724 (<0.001) | 0.599 (<0.001) | 0.584 (<0.001) | 0.764 (<0.001) | 0.767 (<0.001) | 0.646 (<0.001) | 0.638 (<0.001) |
| s20 | 0.426 (<0.001) | 0.469 (<0.001) | 0.235 (0.002) | 0.167 (0.028) | 0.439 (<0.001) | 0.573 (<0.001) | 0.344 (<0.001) | 0.271 (<0.001) |
| s21 | 0.582 (<0.001) | 0.278 (<0.001) | 0.048 (0.522) | 0.110 (0.136) | --- | --- | --- | --- |
| s22 | 0.509 (<0.001) | 0.286 (<0.001) | 0.065 (0.374) | 0.082 (0.260) | 0.470 (<0.001) | 0.214 (0.003) | -0.004 (0.951) | 0.022 (0.763) |
| Mean Fisher Z | 0.662 (---) | 0.558 (0.034) | 0.305 (<0.001) | 0.320 (<0.001) | 0.622 (---) | 0.566 (0.175) | 0.339 (<0.001) | 0.342 (<0.001) |

Strength of correlation indicated by Pearson’s correlation coefficients between ∆CBFv and RGE metrics including bER, ∆PO_2_, ∆PCO_2_ and P_ET_CO_2_ (n=13). Numbers in brackets next to Pearson’s correlation coefficients indicate p values from individual correlation analyses. The bottom row shows the mean values of Fisher’s Z scores transformed from Pearson’s correlation coefficients in groups. Numbers in brackets next to mean Fisher’s Z scores indicate p values in the paired comparisons.
